# Supplementary material for: Lithium-ion battery components are at the nexus of sustainable energy and environmental release of per- and polyfluoroalkyl substances
Source: Nat Commun. 2024 Jul 8;15:5548. doi: 10.1038/s41467-024-49753-5 (PMC11231300; doi:10.1038/s41467-024-49753-5)
Supplement: Supplementary file 1 — Supplementary Information [file 41467_2024_49753_MOESM1_ESM.pdf]

Supplementary Information for

Lithium-ion battery components are at the nexus of sustainable energy and environmental release of per- and polyfluoroalkyl substances

Jennifer L. Guelfo, P. Lee Ferguson, Jonathan Beck, Melissa Chernick, Alonso Doria-Manzur, Patrick W. Faught, Thomas Flug, Evan P. Gray, Nishad Jayasundara, Detlef R. U. Knappe, Abigail S. Joyce, Pingping Meng, Marzieh Shojaei
Corresponding authors: jennifer.guelfo@ttu.edu; lee.ferguson@duke.edu

1. Department of Civil, Environmental, and Construction Engineering, Texas Tech University, Lubbock, TX, USA
2. Department of Civil and Environmental Engineering, Duke University, Durham, NC, USA
3. Nicholas School of the Environment, Duke University, Durham, NC, USA
4. Archer Science, Lake Elmo, MN, USA
5. Department of Civil, Construction, and Environmental Engineering, North Carolina State University, Raleigh, NC, USA
6. Department of Chemistry, Eastern Carolina University, Greenville, NC, USA

† These authors contributed equally to this work
*Department of Civil, Environmental, and Construction Engineering, Texas Tech University, Box 41023, Lubbock, TX 79409, USA Box 41023; email: jennifer.guelfo@ttu.edu
*Department of Civil and Environmental Engineering, Duke University, Wilkinson Building Room 232, Durham, NC 27705; email: lee.ferguson@duke.edu

The PDF file includes:

Supplementary Notes 1 to 9
Supplementary Figures 1 to 23
Supplementary Tables 1 to 8
Supplementary References

Other Supplementary Materials for this manuscript include:

Supplementary Data 1 to 32 (single Excel file, Guelfo et al. Supplementary Data S1-S32_v2.)
A zip file (Track density raw data - Full set) with *D. Magna* swimming track density data arranged by exposure dose.

Supplementary Notes

Supplementary Note 1: Background

There is no universal definition of PFAS, but there are three commonly referenced definitions[1,2]. The Organisation for Economic Co-operation and Development (OECD) defines a PFAS as, "fluorinated substances that contain at least one fully fluorinated methyl or methylene carbon atom[1]." Additionally, a landmark paper by Buck et al. defined perfluoroalkyl PFAS as, "aliphatic substances for which all of the H atoms attached to C atoms... have been replaced by F atoms...[2]." The United States Environmental Protection Agency (USEPA) recently added PFAS as a class to a list of unregulated contaminants that will be monitored in drinking water across the United States[3]. For this purpose, PFAS are defined as chemicals that have at least one of R-(CF2)-CF(R')R", R-CF2OCF2-R', or CF3C(CF3)RR', and USEPA has provided a list of 10,239 PFAS that meet this definition on the USEPA Comptox dashboard[4].

Bis-perfluoroalkyl sulfonimide (bis-FASI) salts including bis-FMeSI and its longer-chain homologues (e.g., bis(pentafluoroethylsulfonyl)imide; bis-FEtSI; **Supplementary Figure 1**) are produced internationally by companies including 3M and Solvay, accounting for over 57% and 13%, respectively, of global production[5–7]. For example, 3M was issued a patent for bis-FASIs in 1999[8], and advertises the Li^+ salt of bis-FMeSI as lithium ion battery (LiB) electrolyte HQ-115[9], manufactured at facilities in Cottage Grove, MN, Cordova, IL, and Antwerp, Belgium[10]. Solvay markets this same compound as LiTFSi[11] and production is centered in Salindres, France[12]. In 2013, Solvay issued a press release reporting that they had doubled their production capacity of the Li salt of bis-FMeSI[13]. Arkema markets a similar electrolyte, lithium bis(fluorosulfonyl)imide (i.e., LiFSi) that has only sulfur-fluorine bonds[7], but also holds patents for battery electrolytes comprised of mixtures of Li salts that include both LiFSi and Li^+ salts of bis-FMeSI[14]. Further, Arkema manufactures Kynar PVDF for use in applications including LiBs[15]. Of the bis-FASIs, bis-FMeSI is most well documented for use in LiB applications, but other perfluoroalkyl homologues including bis-FEtSI (CAS 132843-44-8) and bis(nonafluorobutylsulfonyl)imide; CAS 119229-99-1; bis-FBSI) are mentioned in LiB electrolyte patents[16], and may be present as unintentionally-produced byproducts of bis-FASI salts[2]. Other lesser uses for bis-FASIs are documented in the literature including use as corrosion inhibitors[17], in hydrosilylation[18], hydroformylation[19], carbon dioxide capture[20], and electrolytes in dye-sensitized solar cells[21]. Notably, these other uses often involve bis-FMeSI with counterions other than lithium, including organic counterions.

There are limited prior reports of bis-FMeSI in surface water and riverbank filtration samples[26,27]. Neuwald et al. (2022) detected bis-FMeSI in 9 samples at concentrations of ≤ 2 ng/L (median 0.8 ng/L) in surface water and riverbank filtration samples[26], and Wang et al (2023) detected bis-FMeSI in 3 sea water samples at concentrations of 0.296-1.5 ng/L[27]. There are no reports of bis-FMeSI in soil or sediment, and no reports of bis-FMeSI homologues such as bisperfluoroethanesulfonimide (bis-FEtSI) and bisperfluorobutanesulfonimide (bis-FBSI) in any environmental media. These prior studies, which referred to bis-FMeSI as NTF2, note the urgent need for additional bis-FASI investigation. Wang et al. (2023) noted that, "…further field data on the environmental behavior of NTF2 is urgently needed[27]." In reference to bis-FMeSI, Neuwald et al. (2022) stated, "Currently, the lack of occurrence data makes it impossible to evaluate if its use in energy storage leads to its environmental release[26]." Conclusions by these authors further highlight the novelty and immediacy of our study objectives and results. Importantly, we will demonstrate detections of bis-FMeSI three orders of magnitude higher than those of previous

studies, the first bis-FMeSI detections in soil and sediment, and the first detections of bis-FBSI or bis-FEtSI in any environmental media.

Occurrence of bis-FMeSI in drinking water in other regions is unknown, but the United States Environmental Protection Agency (USEPA) released a chronic reference dose (RfD) of 0.3 $\mu g/kg_{bw}$-day for bis-FMeSI based on studies in a European Chemicals Agency (ECHA) bis-FMeSI dossier[22,23]. This RfD was rated as "low confidence" by USEPA based on concerns with the quality of the underlying data[22]. Nevertheless, the USEPA RfD points to increased regulatory scrutiny of bis-FMeSI exposure and an associated need to understand treatability. A single study of powdered activated carbon treatment at a wastewater treatment plant (WWTP) found no measurable removal of bis-FMeSI, but both influent and effluent bis-FMeSI concentrations (~0.8 ng/L) were ~50% of the limit of quantitation (1.5 ng/L)[24]. Further assessment of bis-FASI removal using adsorption (e.g., activated carbon, ion exchange), and advanced treatment approaches (e.g., oxidation) is warranted.

PFAS releases have occurred from primary manufacturers of PFAS (e.g., Chemours, Parkersburg, WV)[25] and from sites where PFAS are used during the manufacturing process (e.g., ChemFab performance plastics in Bennington, VT)[26]. For example, atmospheric emissions from secondary manufacturer ChemFab leached into groundwater after surface deposition and caused perfluorooctanoic acid (PFOA) concentrations up to 600 ng/L in public supply and residential wells over an area >200 km^2[26]. By the time this release was discovered in 2015, use of PFOA was phased out, but it had been replaced with other PFAS including perfluoro-2-propoxypropanoic acid (HFPO-DA; trade name GenX). Information about replacement PFAS is considered confidential business information in the US, so releases of compounds such as HFPO-DA were initially discovered through monitoring efforts in the Cape Fear River near a Chemours primary manufacturing facility[27]. This led to subsequent discovery of concentrations of HFPO-DA up to 631 ng/L in raw water at a drinking water treatment plant in the same watershed[28]. Collectively, these studies demonstrate potential for widespread environmental impacts of PFAS as a result of both primary and secondary manufacturers, and the value of independent monitoring of manufacturing sites to elucidate undisclosed releases of PFAS. Primary manufacturers have a documented history of failing to disclose information on the human health impacts of PFAS,[29] which adds a layer of importance to independent studies of PFAS occurrence as well as toxicity and treatability.

Daphnia magna (*D. Magna*) have been used to evaluate the toxicity of individual toxicants and effluent wastes for more than 90 years[30]. These organisms are excellent indicators of toxicity because they are sensitive to low concentrations of different toxicants (1mg/L – 100 µg/L)[31]. Further, they are easily maintained in lab cultures, reproduce asexually, eliminating genetic variability in the test population, and are a representative species at the bottom freshwater food chains[31]. Most published *D. magna* toxicity testing data uses lethality as the main test endpoint[31,32]. However, there has been a paradigm shift from lethality based toxicity testing to developing sublethal methods capable of identifying effects at environmentally relevant exposures[33]. *D. magna* are an excellent candidate for sublethal toxicity testing as there are currently 48 sublethal effects identified across four classes (reproduction, swimming behavior, biochemical, and physiological changes)[31]. Perturbations to *D. magna* swimming behavior as a result of toxicant exposure is a consistently sensitive endpoint class with respect to dose[32].

Many of the sublethal endpoints identified in *D. magna* were identified using exposures to a variety of pharmaceuticals (e.g. antibiotics, beta blockers)[31]. Effects to swimming parameters have been observed as low as 500 ng/L. This effect level underscores the utility of *D. magna* as a

sensitive indicator of toxicity[34]. Further, this growing body of pharmaceutical effects data allow conclusions to be drawn between known human mechanism of action and the effects observed in D. magna. Compared to pharmaceuticals, there is a limited knowledge of the toxicity of PFAS to *D. magna*, none of which evaluate sublethal effects on swimming. Therefore, the aim of this work is to evaluate the effects of bis-FMeSI on *D. magna* at environmentally relevant concentrations reported in this study.

Zebrafish larval locomotion is a widely used method for identification of neurobehavioral effects and an indicator of sub-lethal and sub-teratogenic toxicity resulting from chemical exposure. Behavioral alterations have been reported in zebrafish larvae at non-teratogenic concentrations of several types of PFAS[35–38]. Chemical exposure has been shown to alter cellular energy metabolism directly by causing mitochondrial dysfunction. Mitochondrial function is also considered to be a biomarker for energy metabolism, and it is one that can be studied in a vertebrate, whole organism by use of embryonic zebrafish. While behavioral alterations have been shown following exposure to PFAS of a variety of structural subclasses, mitochondrial effects are considerably less well studied for these types of compounds particularly in zebrafish. Hagenaars et al. (2013) reported PFOA caused an increase in mitochondrial permeability as well as a decrease in electron transport chain activity likely resulting from a decrease in ATP production[39]. Similar evaluations of bis-FASIs are not available.

Prior studies of PFAS occurrence and toxicity indicate a need for effective PFAS treatment approaches, but most conventional treatment approaches for media such as drinking water are ineffective for complete removal of PFAS[40,41]. Highly recalcitrant PFAS such as PFOA, perfluorooctanesulfonic acid (PFOS), and their homologues are not readily mineralized during oxidation[42]. Under some conditions, concentrations of perfluoroalkyl carboxylates (PFCAs; i.e., PFOA and homologues) may increase during oxidation because they are terminal daughter products of oxidizable PFAS[42]. Even PFAS that can be degraded during oxidation are recalcitrant because their terminal daughter products are still PFAS. Since oxidative approaches are routinely employed for disinfection during treatment, it is important to understand how PFAS may behave in these systems. Although removal is unlikely, parent PFAS may have different properties than their terminal oxidative transformation products,[43] which may impact the approach used to remove those products during subsequent treatment steps (e.g., adsorption). Researchers are investigating destructive techniques for PFAS,[44] but adsorption-based removal using with granular activated carbon (GAC) and/or ion exchange (IX) resin is more readily implemented in full-scale systems[45]. Collectively, screening novel PFAS for fate during oxidation and adsorption-based treatment will inform their recalcitrance and treatability relative to PFAS for which there are already regulatory drivers for removal (e.g., PFOA, PFOS).

Supplementary Note 2: Field blanks and duplicates

PFAS in all project field blanks were below detection with the exception of 1.06 ng/L GenX detected in January 2022 samples (**Supplementary Data 1**). PFAS concentrations in aqueous field duplicates were generally within 25% (**Supplementary Data 1, 2,** and **6**), which indicates sampling protocol collected representative samples. There were limited exceptions when evaluating results of duplicate samples in the single digit ng/L levels where even minor concentration differences comprise percentages > 25% (e.g., PFOS concentrations of 7.63 and 4.90 ng/L in duplicate samples from EU 9, **Supplementary Data 6**).

Supplementary Note 3: Minnesota results in the Minneapolis-St. Paul region

In both January and June, there were unexpected detections of comparatively low concentrations (≤12 ng/L; **Supplementary Data 1** and **2**) of perfluoroalkyl ether acids (PFEAs) in samples proximal to 3M plant discharge (MN 4, MN 22), even though 3M is not a known producer of PFEAs. It is possible that 3M processes that use hexafluoropropylene oxide are at least partially responsible for unintentional production of PFEAs[46]. Regardless, these results demonstrate that environmental occurrence of PFEAs may result from manufacturers other than e.g. Chemours[28], albeit at much lower levels.

Supplementary Note 4: Minnesota results in the Lake Elmo region

June sampling included locations north of Cottage Grove in Lake Elmo, MN, which has documented PFAS impacts resulting from historical disposal of 3M waste in the former Washington County Landfill (1969-1975). Concentrations in surface waters near Lake Elmo were <1 ng/L with the exception of 4.54 ng/L detected in Lake Elmo (MN 27). Due to relatively low concentrations, it is unclear if this results from legacy PFAS disposal or dispersed, atmospheric deposition. The oldest 3M patent that could be located for bis-FASIs was submitted in 1995, but presumably research and development pre-dated 1995. Lake Elmo is situated northeast of 3M Cottage Grove and crosswind of the primary wind directions (**Fig. 1,** main manuscript). Regardless of source and as noted in the main manuscript, these results demonstrate wide distribution of bis-FMeSI aqueous impacts in the Minneapolis-St. Paul region.

Supplementary Note 5: Minnesota results Avian species observations

Multiple avian species were observed near the Mississippi River sampling sites including *Haliaeetus leucocephalus* (bald eagle), *Cygnus buccinator* (trumpeter swan), and *Anas platyrhynchos* (mallard) (**Supplementary Figure 9**). During January samples, birds were observed near unfrozen portions of the river such as warmer water in the vicinity of the 3M outfall (MN 4). While the toxicity of PFAS to these bird species is not well understood, a recent study proposed chronic reproductive toxicity thresholds for avian species (*Colinus virginianus* or bobwhite quail) of 100 ng/L via drinking water. These values decreased to <100 ng/L when mixtures were present, suggesting potential for synergistic effects[47]. A recent study of PFAS in bald eagle nestlings from 3 industrialized areas found that PFAS were most elevated in the region of the 3M Cottage Grove facility[48]. Collectively results of this study and others raise significant questions regarding the impacts of aqueous releases of bis-FMeSI and other PFAS on terrestrial organisms in the region.

Supplementary Note 6: Europe results

Detections of bis-FASIs in the Antwerp region are discussed in the main manuscript. A total of 30 additional PFAS were detected at concentrations of 1.16 ng/L (PFO4DA) to 145,000 ng/L (PFOS; **Supplementary Data 6**). PFBA (1.21-40,700 ng/L), PFPeA (5.74 - 36,700 ng/L), PFHxA (12.7 - 94,000 ng/L), PFHpA (3.81 - 40,800 ng/L), PFOA (5.18 - 68,200 ng/L), PFBS (5.38 - 20,900 ng/L), and PFOS (5.02 - 145,000 ng/L) were detected in all surface water samples. PFEA concentrations up to 178 ng/L (PFMOAA) were observed at EU 17, consistent with aqueous PFEA detections at MN 4 near the 3M, Cottage Grove facility. In Antwerp soils and sediments, 27 additional PFAS were detected at concentrations of 2.08 (EtFOSAA, EU 19 soil) - 23,040,288 (PFOS, EU 17 sediment) ng/kg. PFCAs (C6, C7, and C9-C14), PFSAs (C8 and C9), and EtFOSAA were detected in all soil and sediments along with more intermittent detections of FASAs (e.g.,

FHxSA and MeFOSAA) and 8Cl-PFOS, which are all consistent with 3M synthesis of PFAS using electrochemical fluorination[2,49]. Fluorotelomer (FT) -based PFAS (e.g., 4:2 FTS and 6:2 FTS) were also detected, but at much lower levels (21.6 - 7,282 ng/kg).

In Salindres, bis-FMeSI was detected at concentrations of 4.36-6.55 ng/L in 2 samples (EU 24 and EU 25) collected in the L'Arias River proximal to and downstream of a Solvay facility (**Supplementary Data 6**). A total of 18 additional PFAS (1.73 ng/L PFDA - 65.3 ng/L PFPeA) were detected in surface water samples from this region, and in the majority of PFAS, maximum concentrations were also detected at EU 24 and EU 25 (**Supplementary Data 6**). Similarly, bis-FMeSI and/or homologues were also detected in all soils and sediment samples collected in the Salindres region at total bis-FASA concentrations of 42.1 (EU 21) - 253 (EU 20) ng/kg and 50.4 (EU 23) - 3,886 (EU 22) ng/kg, respectively, and bis-FBSI was the dominant homologue in 8 of 11 samples with bis-FASI detections (**Supplementary Figure 14, Supplementary Data 7**). Despite similar bis-FASA distributions in the soils and sediments of the Antwerp and Salindres regions, signatures of other PFAS were reflective of different production chemistry. A total of 23 additional PFAS were detected at concentrations of 13.0 (PFHxS, EU 25 sediment) - 40,433 (PFOS, EU 22 sediment) ng/kg, so maximum concentrations were orders of magnitude lower than in Antwerp, and maximum FT-based PFAS concentrations (e.g., 13,952 ng/kg 6:2 FTS in EU 22 sediment and 11,431 ng/kg 8:2 FTS in EU 20 soil) were the same order of magnitude as PFOS.

Supplementary Note 7: *D.magna* toxicity

Unequal variance that changes with exposure dose is an underutilized but promising indicator of toxicity in exposed populations. Studies suggest it may be an earlier and more sensitive indicator of toxicological effects relative to parameters such as the mean[50–52]. A Chi^2 test with a Bonferroni correction was used as an intercomparison metric to assess differences in velocity variance of control organisms vs. variance at each exposure concentration. Velocity variance at doses of 10, 1000, and 5000 ng/L bis-FMeSI were different from control variance.

As noted in the main manuscript, there is recognition in the literature that heterogeneity of variance can be an earlier and more sensitive indicator of toxicological effects relative to changes in the mean[50–53]. Studies attribute changes in variance to experimental factors (e.g., analytical variability), genetic variability, and non-genetic phenotypic response.[50] In this study, bis-FMeSI exposure concentration was the only change in experimental condition, and *D.magna* are genetically identical to each other. Thus, changes in variance of a monitored endpoint relative to the control are attributable to changes in exposure concentration. As noted above, changes in variance are evaluated by comparing variance at each exposure level to the control using a Chi^2 test. Because variance is heterogenous, criteria for use of ANOVA (i.e., equal variance, normal data distribution) are not met, so there are no comparisons between doses. Studies have identified that differences in variance relative to controls may occur only in some exposure concentrations (e.g, **Fig. 2** of the manuscript), but still recognize heterogenous variance as an early toxicological indicator[52].

In this study, heterogenous variance indicates that exposure to bis-FMeSI has a significant effect on the variance of swimming velocity at concentrations as low as 10 ng/L. The means of swimming velocity did not differ significantly, but the outcomes of variance testing still demonstrate an effect of exposure. This is similar to prior *D.magna* studies that observed no effect of 48-hr contaminant exposure on mean oxygen consumption, but significant changes in the variance of oxygen consumption.[51] Their results,[51] results of additional prior work,[52] and results herein highlight that testing homogeneity of variance has utility beyond its traditional use as

criteria for use of ANOVA. The impacted endpoint (velocity) indicates an effect on swimming behavior, which is controlled in *D.magna* by the central nervous system[54]. Because of this, prior studies have noted that changes in swimming behavior may be indicative of a neuroactive effect[54,55]. Additional study would be required to confirm the mechanism of action of bis-FMeSI exposure.

As noted in the main manuscript, we did not identify prior studies that investigated sublethal impacts of PFAS on *D. magna* swimming behavior. A single study measured an immobilization effective concentration (EC_{50}) of 130 mg/L,[56] which is consistent with our observation that immobilization did not correlate with dose up to the maximum evaluated concentration of 5000 ng/L (**Supplementary Figure 16**). Two prior studies concluded that PFBA, PFHxA, PFOA, PFNA, PFHxS, PFOS, and GenX lead to metabolic perturbations in *D. magnia*, which may be consistent with changes in swimming behavior observed herein; however exposure concentrations were ≥1 mg/L[57,58]. Prior studies with non PFAS toxicants have observed more significant impacts in endpoints evaluated here, but as with PFAS studies, dosing concentrations were higher[54]. These results demonstrate the value of sublethal *D. magna* testing as a method of screening toxicity at the ng/L level, and support further exploration of variance as a novel toxicity endpoint.

The water quality parameters for *D. magna* toxicity test media are reported below in **Supplementary Table 6**. All measured data are meet the required values or ranges described in EPA-821-R-02-012[59]. The nominal concentrations of bis-FMeSI are also reported in **Supplementary Table 6**, and increase from lowest highest exposure concentration.

Supplementary Note 8: Zebrafish toxicity

There were no differences from controls in any of the concentrations of bis-FMeSI or water samples tested for survival, hatching, or developmental deformities in zebrafish used for mitochondrial and locomotion assays (**Supplementary Figure 17**). In the additional three highest concentrations tested for acute endpoints only, the highest concentration (250,000,000 ng/L) had >80% of larvae with uninflated swim bladders and ~20% of larvae with pericardial and yolk sac edema. Body lengths were the same between larvae in all groups used for mitochondrial and locomotion assays (**Supplementary Figure 18A**). At 6 wpf, body lengths were mostly similar between experimental groups. There were statistically significant differences between fish that had been exposed to 25 ng/L bis-FMeSI compared to control and 25,000 ng/L (**Supplementary Figure 18B**). Likewise, fish in the 250 ng/L were different than those in control. This is very likely due to the lower tank density in 25 ng/L (n=8) and 250 ng/L (n=9) compared to other tanks such as control (n=17). It is well established in zebrafish that fish naturally grow larger when tank densities are lower[60]. Therefore, this is very likely due to this rather than a treatment effect.

The zebrafish assay for mitochondrial function included basal mitochondrial respiration, adenosine triphosphate (ATP) production, non-mitochondrial respiration, proton leak, spare capacity, maximum mitochondrial respiration, and non-mitochondrial respiration. Concentration dependent decreases were observed for all parameters except ATP production and non-mitochondrial basal respiration, with the highest concentration tested (i.e., 250,000 ng/L) statistically significant from the control and/or 25 ng/L exposure (**Supplementary Figure 19**). Decreases in basal respiration indicate that embryos exposed to high concentrations of bis-FMeSI do not produce as much energy in a resting state. These differences are reflected in decreased maximum mitochondrial respiration and spare capacity, which show that these embryos are unable to make up this energy deficit even with an artificial energy demand. Such decreases in energy

may be due to decreased substrate availability or compromised mitochondrial mass or integrity[61–63].

While not statistically significant, it should be noted that there were slight increases in maximal mitochondrial respiration and spare capacity for embryos exposed to the lowest concentration tested in this assay (i.e., 25 ng/L). This trend is consistent with the hyperactivity observed in the larval locomotion assays (**Fig. 3**, main manuscript). While zebrafish behavioral changes have been reported following exposure to a variety of PFAS, bioenergetics is considerably less well studied. This is interesting because behavior and energy production are, in many ways, linked. Many mitochondrial defects have been shown to affect the nervous system[64,65]. For example, Huang et al. (2023) found that exposure to PFHpA decreased ATP-linked respiration in zebrafish embryos and reduced locomotor activity in larvae[66]. Patel et al. (2022) reported changes in expression levels of mitochondrial-related genes but no changes in mitochondrial function or larval locomotion[67]. The decrease that we observed in both the behavior test as well as the mitochondrial assay suggests that exposure to bis-FMeSI is energetically costly and provides information on potential modes of action of bis-FMeSI. Increases that we observed at the lowest concentrations may be related to more than one mechanism (e.g., bioenergetics and neurological function). Additional experiments are needed to resolve these questions.

Supplementary Note 9: Oxidative Treatment

The European Commission's (EC's) recently proposed addition of a very persistent very mobile (vPvM) hazard class to multiple regulations[68]. Under the vPvM paradigm, biodegradation ½-life is used to evaluate persistence and mobility is evaluated using the organic carbon-water partitioning coefficient (K_{oc})[68]. Specifically, compounds with log $K_{oc} < 2.0$ and biodegradation ½-life > 180 days meet criteria for designation as vPvM. Field K_d values for bis-FMeSI were similar to PFPeA and PFBS (**Supplementary Figure 10**), both of which have log K_{oc} values < 2[69], suggesting that bis-FMeSI is very mobile. As observed for PFOA and PFOS in prior studies[42], bis-FMeSI was recalcitrant under alkaline, oxidative conditions (**Supplementary Figure S23**). Studies have also found that bis-FMeSI does not hydrolyze over a pH range of 1-13 and does not degrade under aerobic activated sludge or anaerobic denitrifying conditions.[70] Although confirmation is needed, collective results of our study and prior work suggest that bis-FASIs will not undergo degradation in the environment, which is also true of PFOA and PFOS[71]. Persistence and mobility data presented here also suggest that bis-FMeSI meets criteria for classification as vPvM under the new EC hazard classification.

The vPvM classification recognizes the potential for compounds to impact aqueous systems over large (e.g., global) regions for extended periods of time, even if production volumes are low relative to other contaminants[68]. In fact, the European Commission has recognized that the concerns about contaminant mobility should be considered equivalent to concerns about bioaccumulation[72]. PFBS is designated as a vPvM compound, and in 2019 it was identified as a substance of very high concern (SVHC) based on its equivalent level of concern (ELoC) to persistent, bioaccumulative, and toxic (PBT) substances[72]. The decision to designate PFBS as a SVHC was based on characteristics including difficulty in removing PFBS during drinking water treatment, continuous exposure as a result of persistence and mobility, accumulation in organisms at an equilibrium level as a result of continuous exposure, high global transport potential based on low volatility, high solubility, and low sorption to soils and sediments[72]. A recent study used systematic case studies to demonstrate that vPvM substances across contaminant classes pose ELoC to PBT substances under REACH[72]. Based on their outcomes, it is reasonable to hypothesize

that bis-FMeSI, which has persistence and mobility similar to PFBS, also poses ELoC as PBT substances. Interestingly, bis-FMeSI also has a similar phospholipid-membrane water partitioning coefficient (log K_{MW}) as PFBS (PFBS log K_{MW}=2.63; bis-FMeSI log K_{MW} = 2.5)[73,74]. Importantly this does not supersede the value in additional assessments of bis-FASI bioaccumulation and toxicity.

Oxidative conditions applied in this study are also used in a sample preparation technique known as the total oxidizable precursor (TOP) assay. The TOP assay is used to quantify the total concentration of unknown oxidizable precursors. It relies on oxidative conversion of unknown PFAS to terminal PFCAs that can be captured during targeted analysis[75]. Increases in PFCAs that cannot be attribute to known oxidizable precursors are often used as a proxy for the total molar concentration of unknown PFAS in a sample.[75] TOP will not capture the concentrations of PFAS that are resistant to oxidation unless they are directly analyzed. Few, if any labs, routinely analyze bis-FASIs, which means their occurrence in samples would not be captured either by targeted or TOP-based analysis using standard methods.

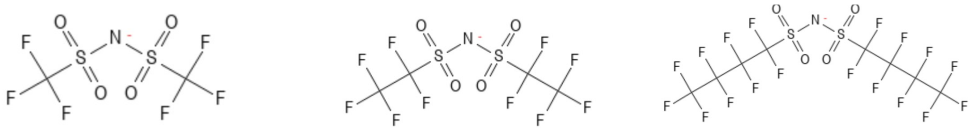

Bisperfluoromethane sulfonimide
Bis-FMeSI

Bisperfluoroethane sulfonimide
Bis-FEtSI

Bisperfluorobutane sulfonimide
Bis-FEtSI

Supplementary Figure 1. Structures of the 3 bis-perfluoroalkyl sulfonimide (bis-FASI) homologues included in this study.

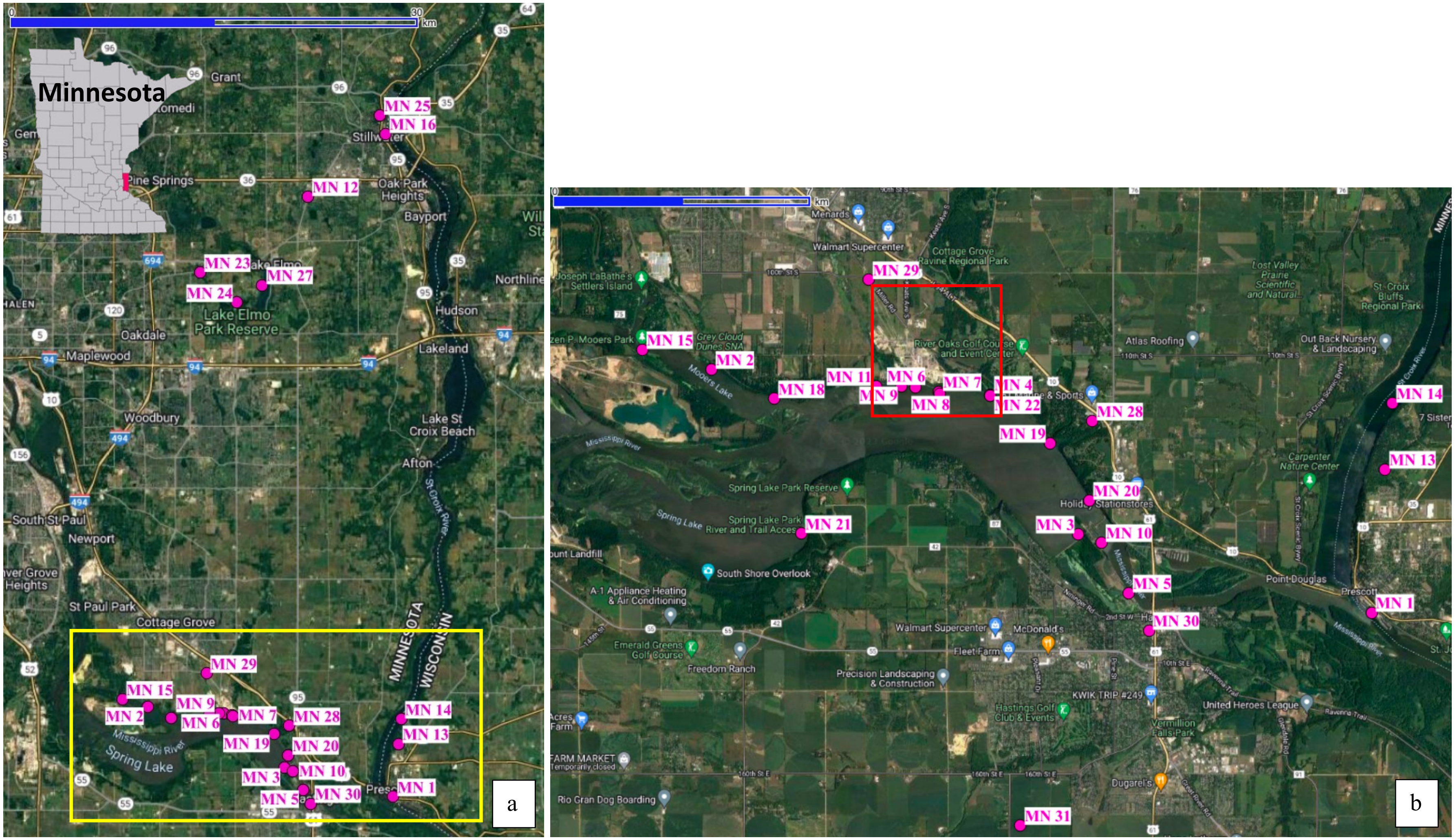

Supplementary Figure 2. Locations of samples collected in January and/or June 2022 near Cottage Grove, MN, US (a). The yellow box is the approximate extent of the map showing the southern sampling locations (b). The red square is the approximate location of 3M, Cottage Grove. MN 17 was collected from an office water bubbler not tied to a geographic location, so it is not depicted. Map data: Google, TerraMetrics 2023. Site location descriptions are in **Supplementary Table 2**.

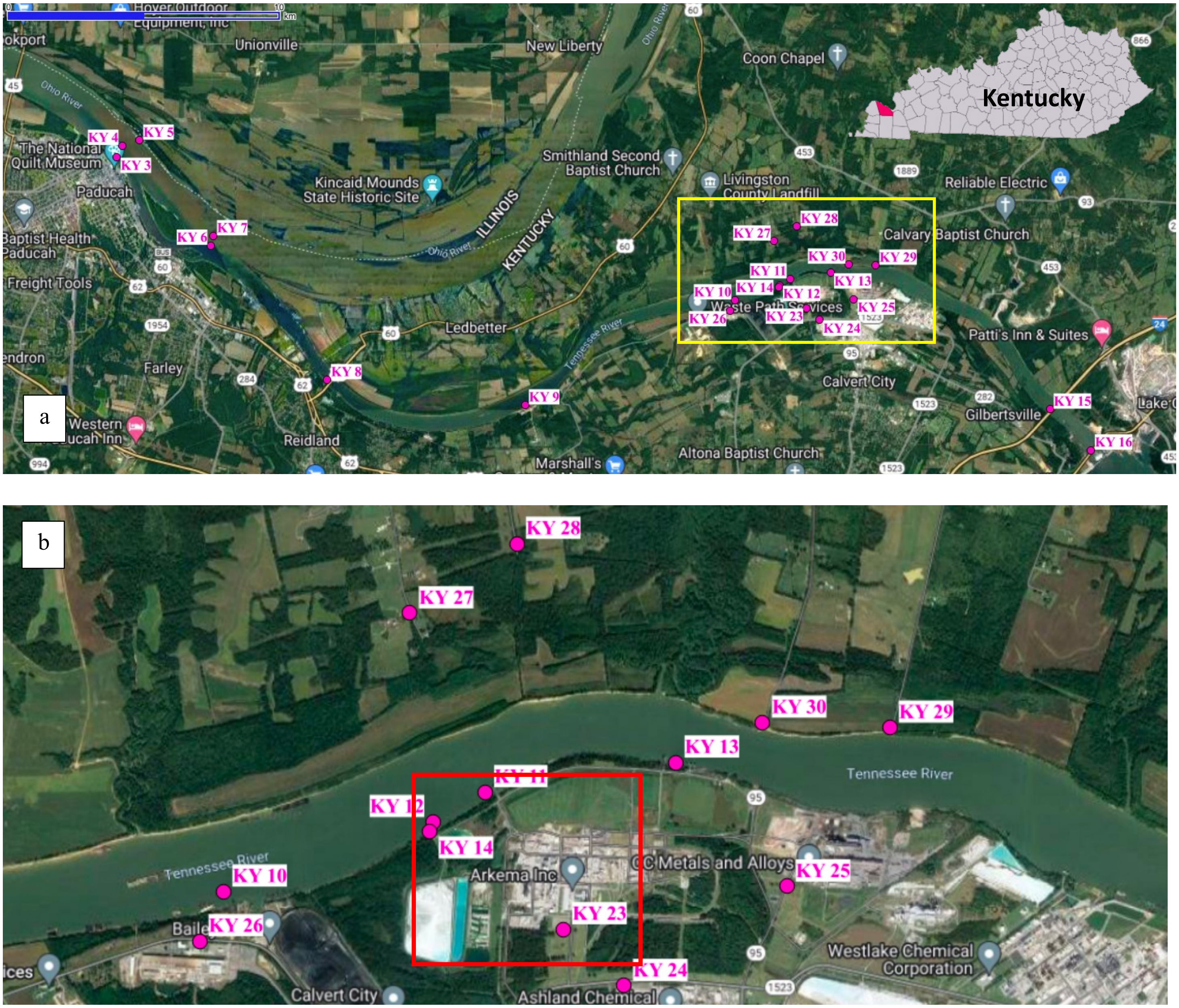

Supplementary Figure 3. Locations of samples collected in September, 2022 near Paducah, KY, US (a). Note that locations KY1 and KY2 were collected while en route to the Paducah region in Knoxville and Nashville, TN respectively, and their locations are not shown. The yellow box is the approximate extent of the central sampling locations (b). The red box is the approximate location of Arkema, Calvert City. Map data: Google, TerraMetrics 2023. Site location descriptions are in **Supplementary Table 3**.

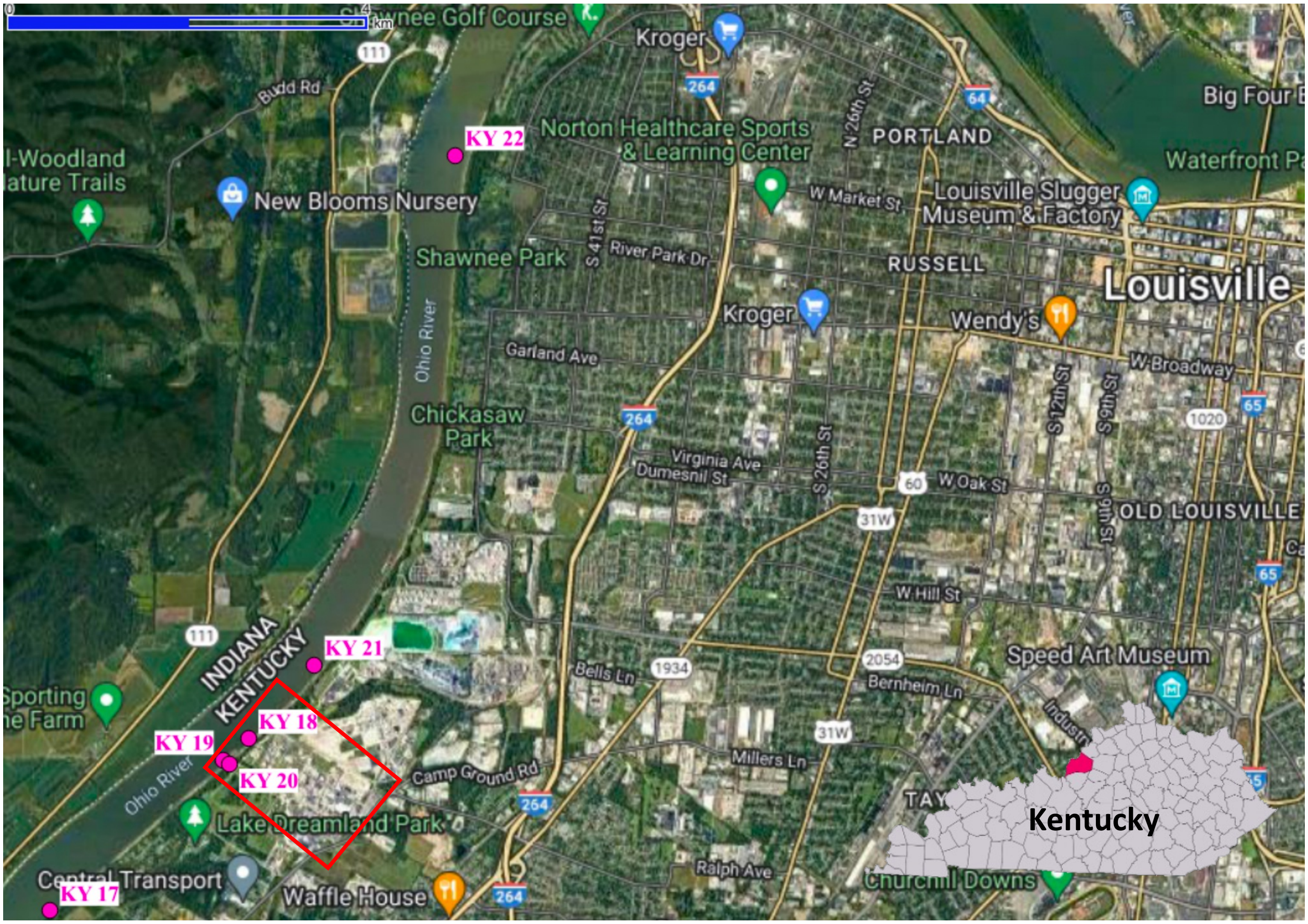

Supplementary Figure 4. Locations of samples collected in September, 2022 near Louisville, KY, US. The red box is the approximate location of Arkema and Chemours in Louisville, KY. Map data: Google, TerraMetrics 2023. Site location descriptions are in **Supplementary Table 3**.

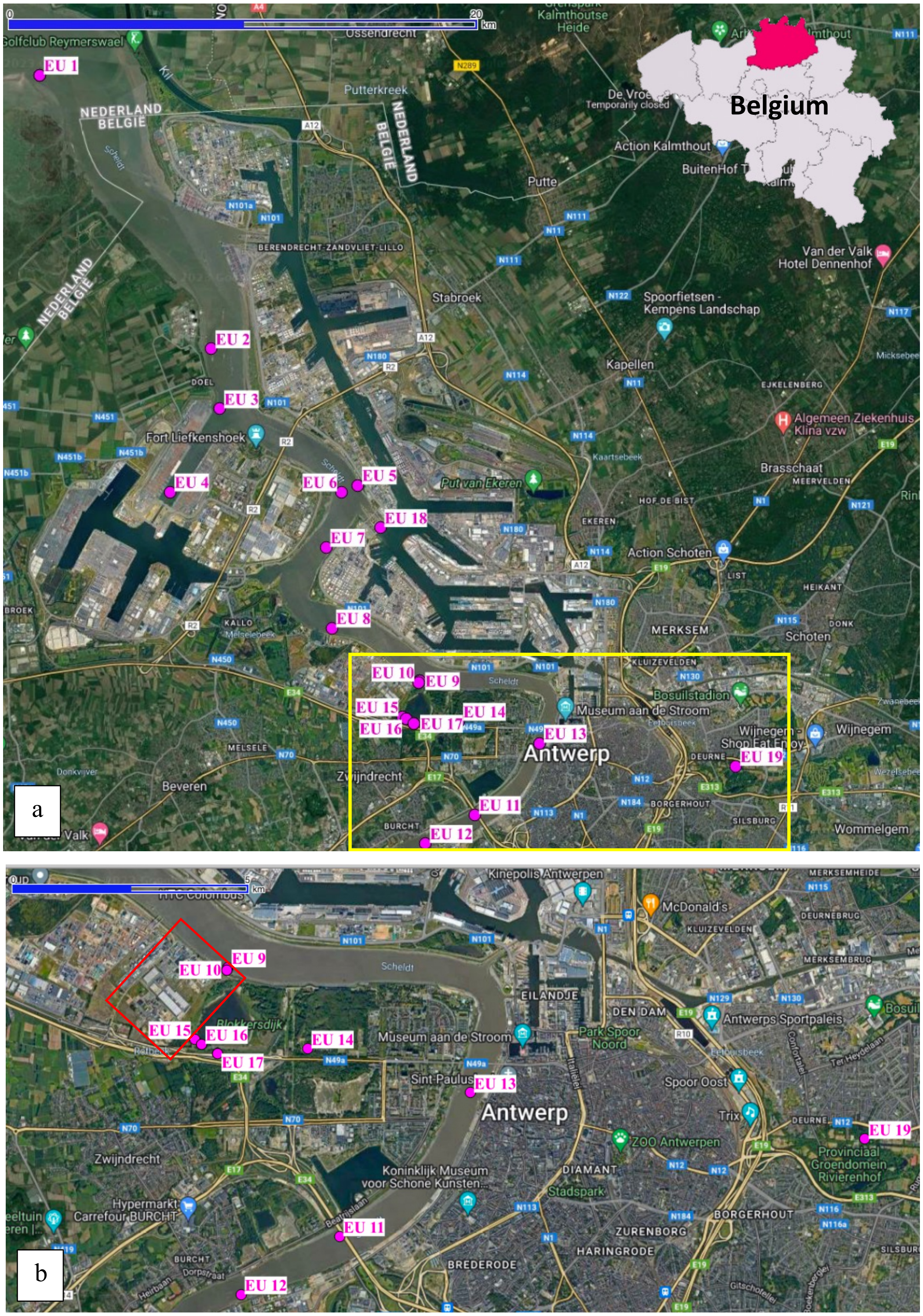

Supplementary Figure 5. Locations of samples collected in October, 2022 near Antwerp, Belgium (a). The yellow box represents the approximate extent of the map showing the southern sampling locations (b). The red square is the approximate location of 3M, Antwerp. Map data: Google, TerraMetrics 2023. Site location descriptions are in **Supplementary Table 4**.

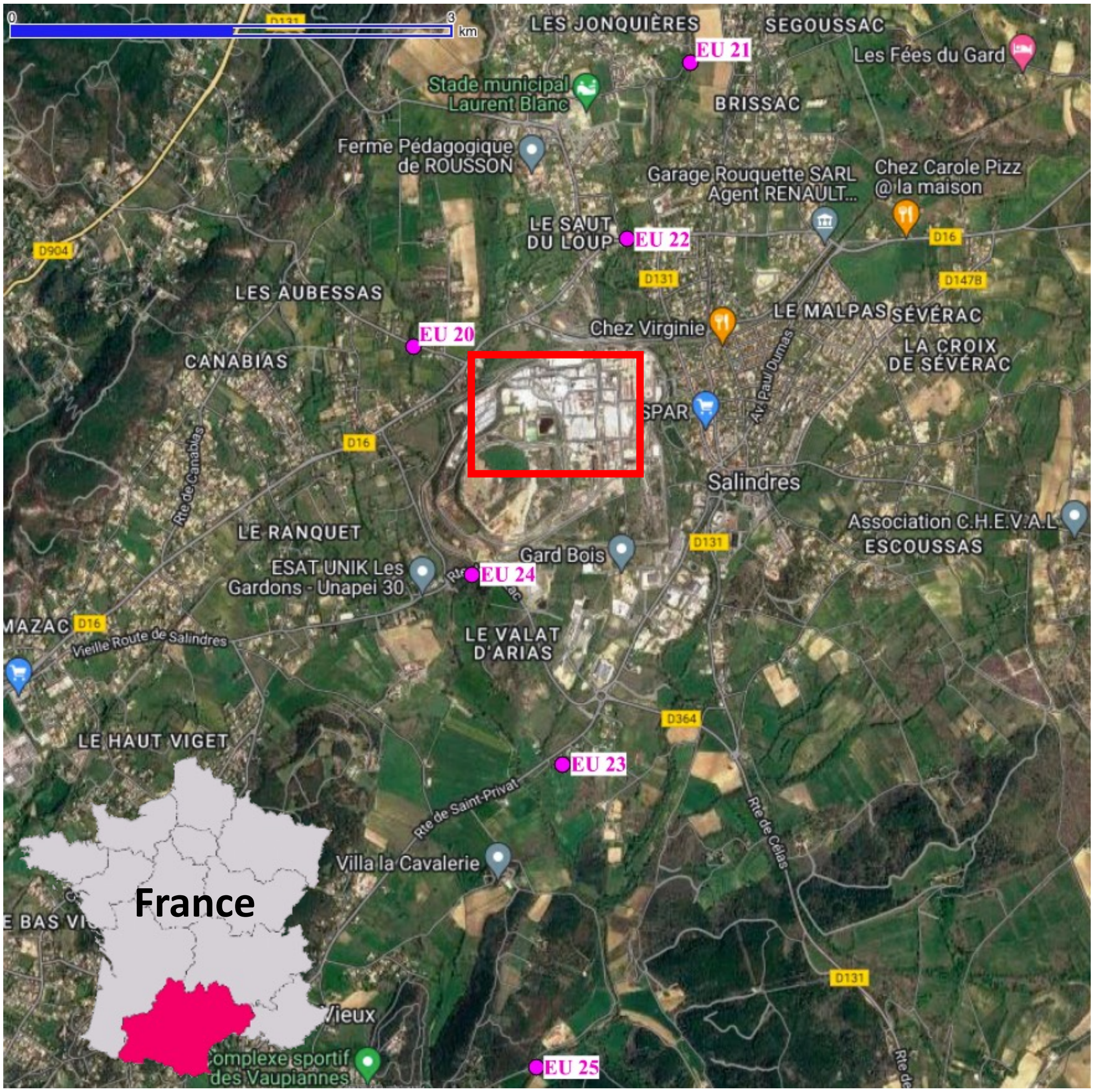

Supplementary Figure 6. Locations of samples collected in October, 2022 near Salindres, France. The red square is the approximate location of Solvay. Map data: Google, TerraMetrics 2023. Site location descriptions are in **Supplementary Table 4**.

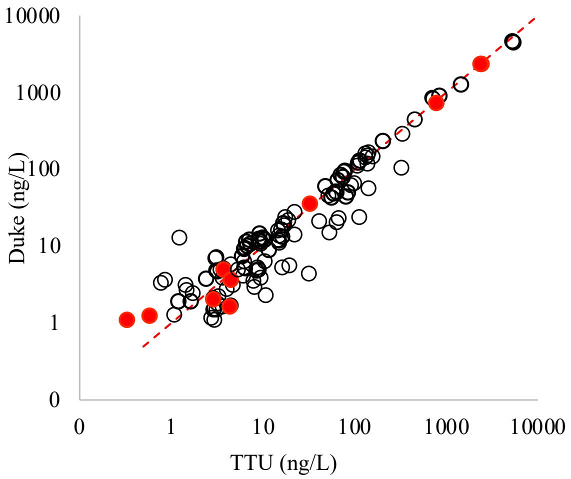

Supplementary Figure 7. A comparison of PFAS concentrations in June aqueous samples from the laboratories at Texas Tech University (TTU; x-axis) and Duke University (y-axis). Red points are bis-FMeSI results, and the red dashed line represents 1 to 1 agreement. Results show excellent agreement between the two labs with the expected increase in variability as results approach the limit of detection. Concentrations reported for the June aqueous samples are in **Supplementary Data 2**.

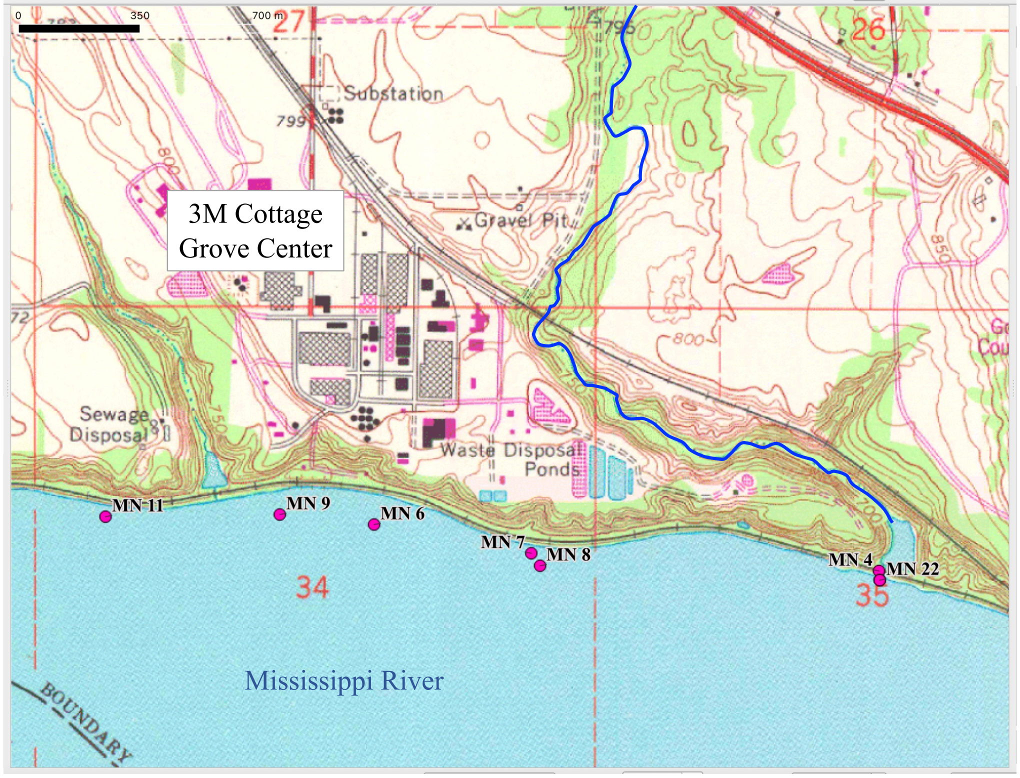

Supplementary Figure 8. United States Geological Survey topographic map showing the location of the 3M Cottage Grove Center and associated disposal ponds. The creek which receives the 3M outfall before flowing into a holding pond and discharging into the Mississippi at sampling location MN 4 has been highlighted **blue**.

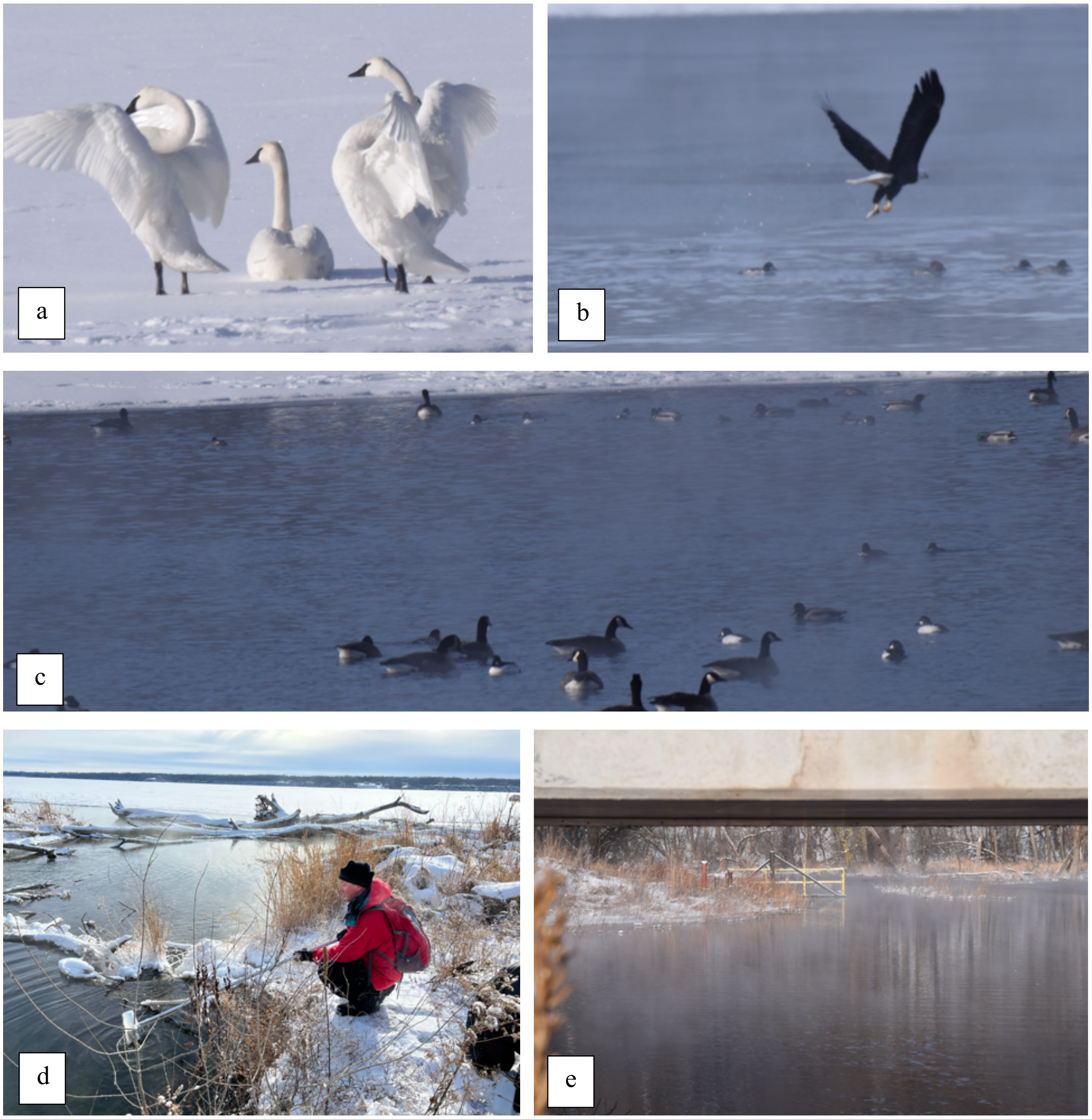

Supplementary Figure 9. Avian species observed in the Mississippi River during the January sampling including *Cygnus buccinator* (trumpeter swan; a), *Haliaeetus leucocephalus* (bald eagle; b), and other species including *Branta canadensis* (Canada Goose; c). Species were observed near unfrozen portions of the river including the 3M outfall creek (MN4) depicted facing towards the Mississippi River during sampling (d) and facing towards 3M Cottage Grove (e).

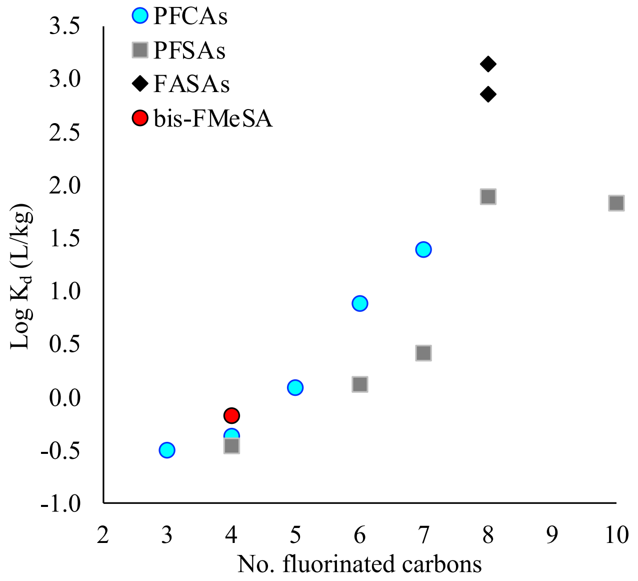

Supplementary Figure 10. Field log K_d values calculated using sediment concentrations (C_s, ng/kg; **Supplementary Data 3**) divided by surface water concentrations (C_w, ng/L; **Supplementary Data 2**) from MN 4 for all per- and polyfluoroalkyl substances (PFAS) that occurred in both media. PFCAs = perfluoroalkyl carboxylates, FASAS = perfluoroalkylsulfonamides, and bis-FMeSI = bis-perfluoromethanesulfonimide.

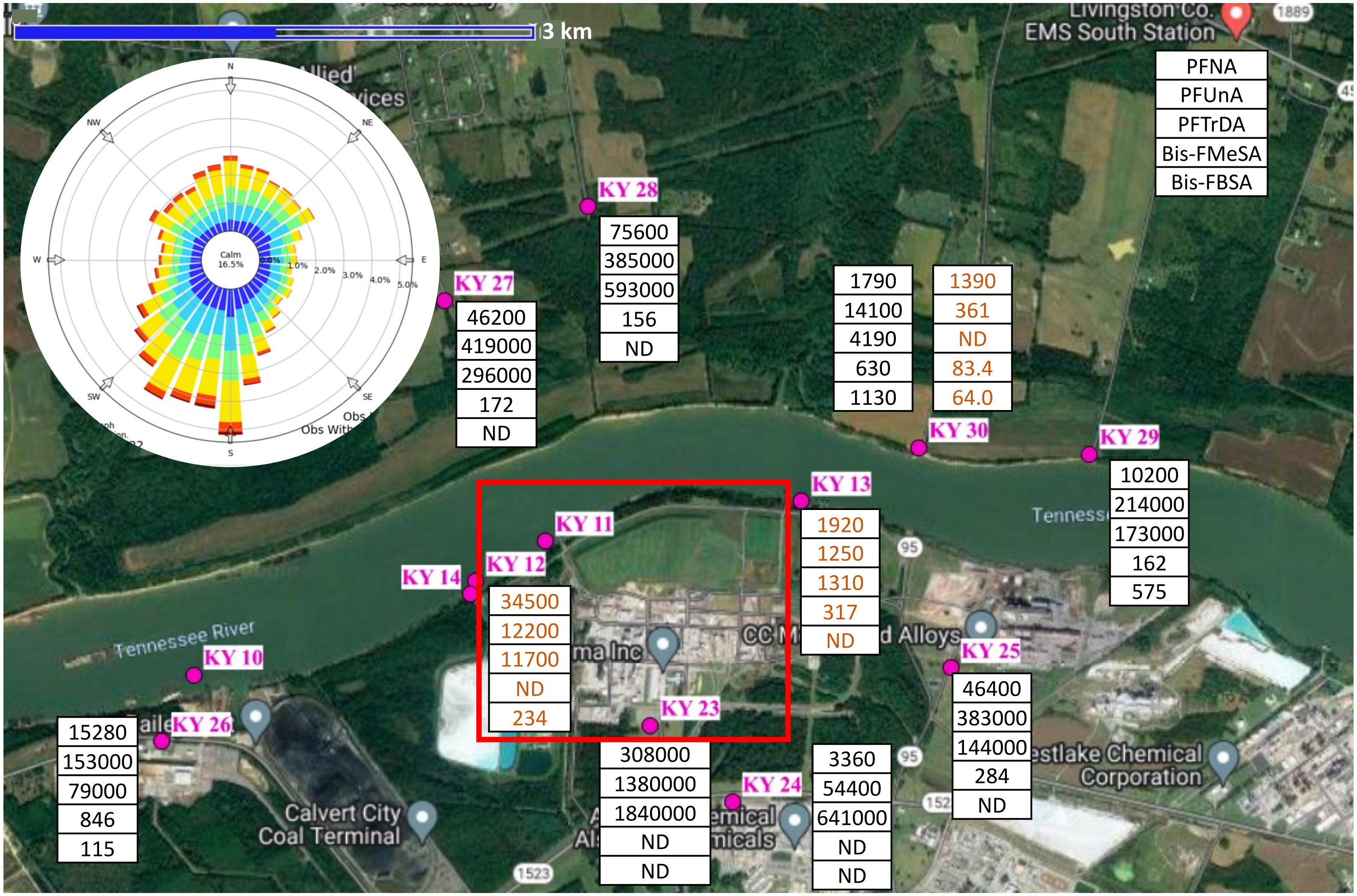

Supplementary Figure 11. Concentrations (ng/kg) of long chain perfluoroalkyl carboyxlates (PFCAs), bis-perfluoromethanesulfonimide (bisFMeSI), and bis-perfluorobutanesulfonimide (bis-FBSI) in soils (black text) and sediment (brown text) collected in and near the Tennessee River in the Paducah, KY region, September 2022. The red box is the approximate location of Arkema facility outside of Paducah in Calvert City, KY. PFNA = perfluorononanoic acid, PFUnA = perfluoroundecanoic acid, PFTrDA = perfluorotridecanoic acid, and ND = non-detect. Source data can be found in **Supplementary Data 5**.

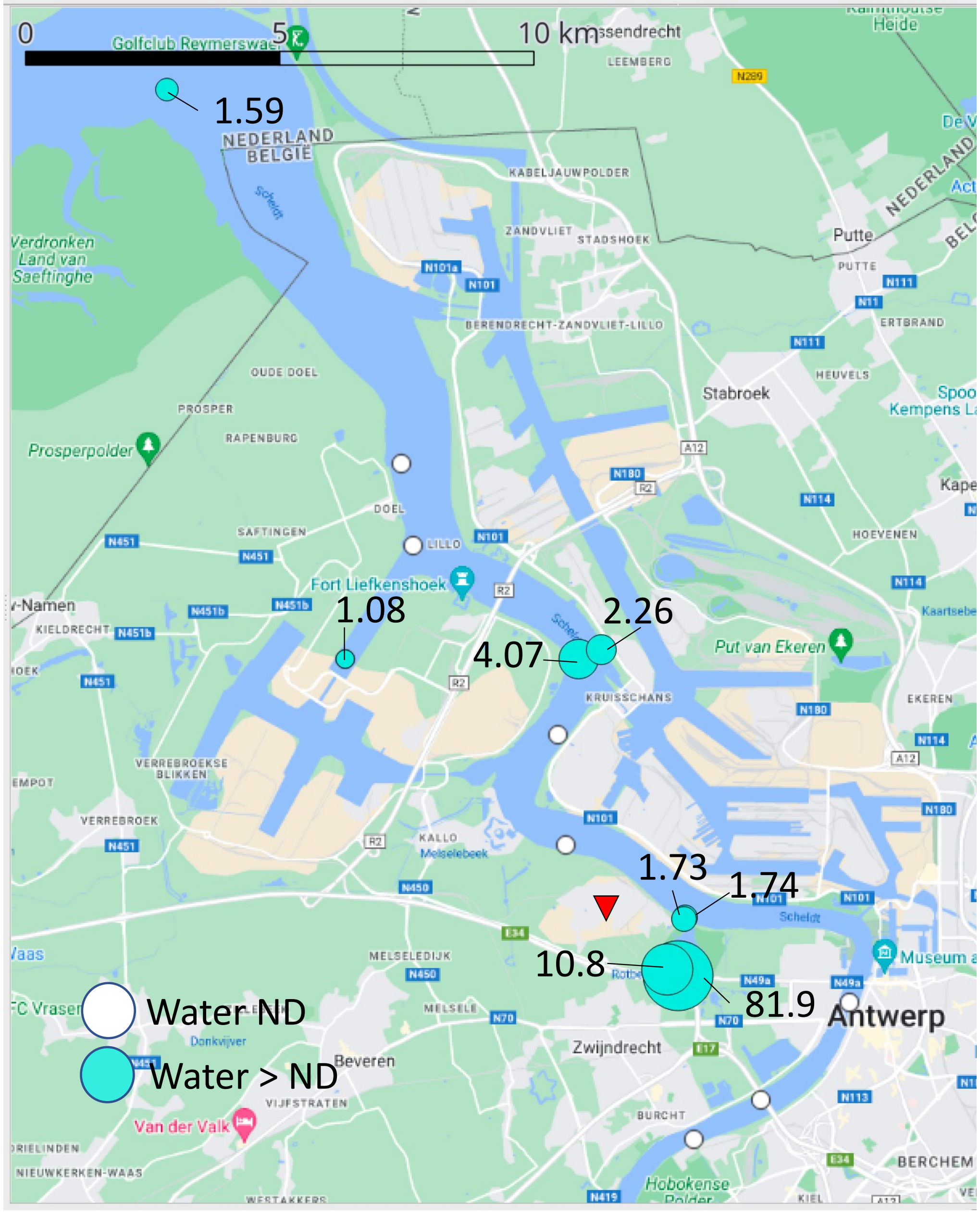

Supplementary Figure 12. Concentrations of bis-perfluoromethanesulfonimide (bis-FMeSI) in surface water (ng/L) in the Antwerp sampling region in October 2022. The red marker denotes the location of 3M Antwerp. ND = non-detect. Source data can be found in **Supplementary Data 6**.

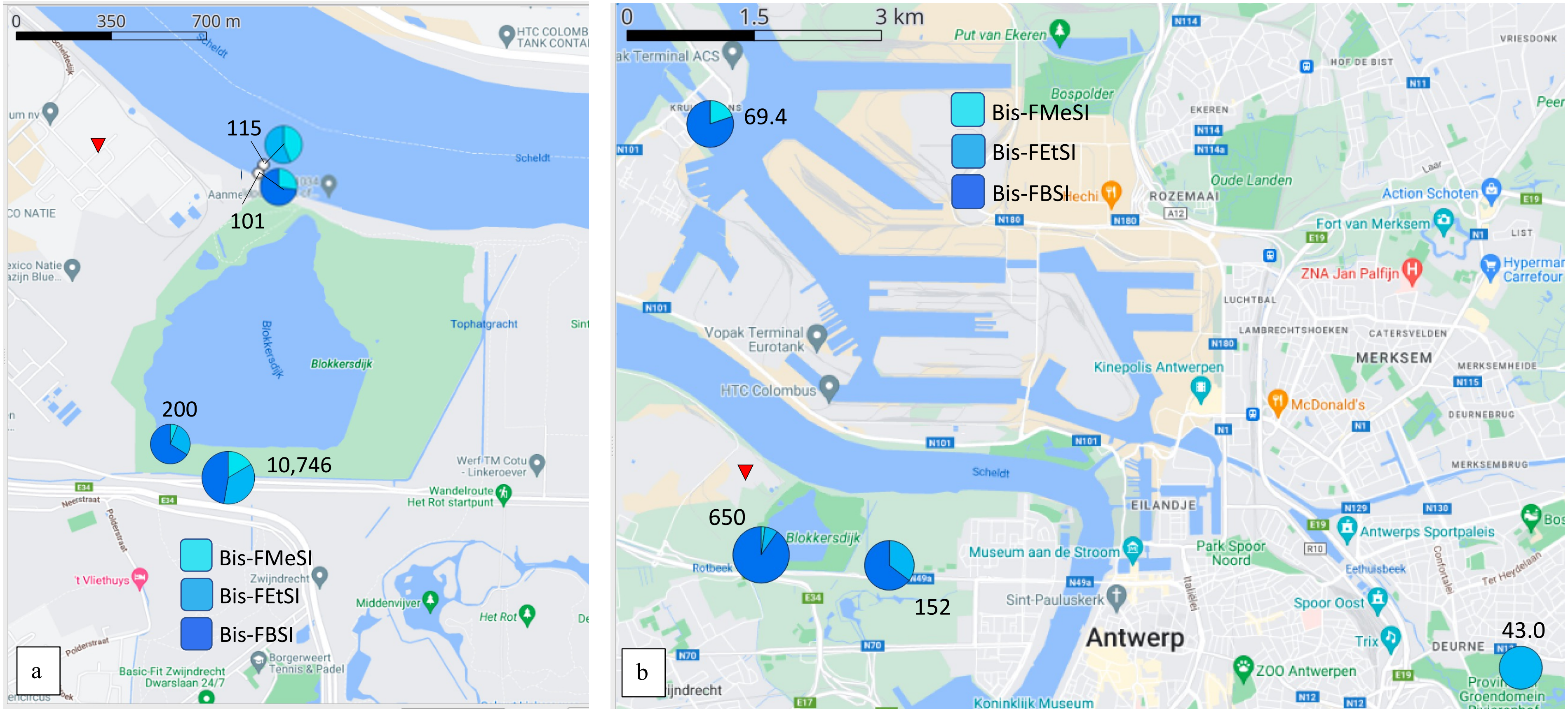

Supplementary Figure 13. Concentrations (ng/kg) of total bis-perfluoroalkyl sulfonimides (bis-FASIs) (sum of bis-perfluoromethanesulfonimide [bis-FMeSI], bis-perfluoroethanesulfonimide [bis-FEtSI], and bis-perfluorobutanesulfonimide [bis-FBSI]) in sediment (a) and soils (b) in the Antwerp region in October 2022 where pie charts denote the fraction of each homologue. The red marker denotes the location of 3M Antwerp. Source data can be found in **Supplementary Data 7**.

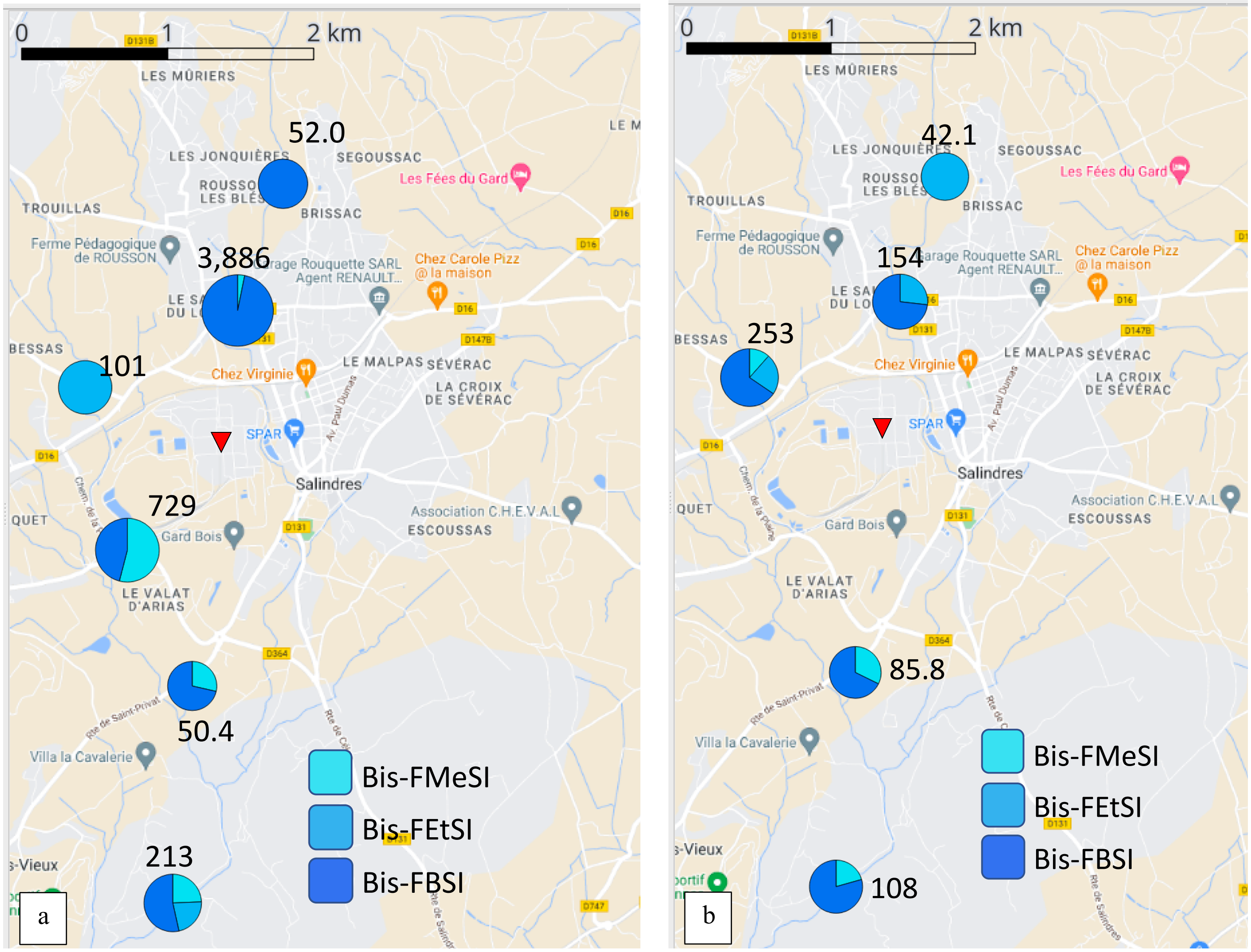

Supplementary Figure 14. Concentrations (ng/kg) of total bis-perfluoroalkyl sulfonimides (bis-FASIs) (sum of bis-perfluoromethanesulfonimide [bis-FMeSI], bis-perfluoroethanesulfonimide [bis-FEtSI], and bis-perfluorobutanesulfonimide [bis-FBSI]) in sediment (a) and soils (b) in the Salindres region in October 2022 where pie charts denote the fraction of each homologue. The red marker denotes the location of Solvay, Salindres. Source data can be found in **Supplementary Data 7**.

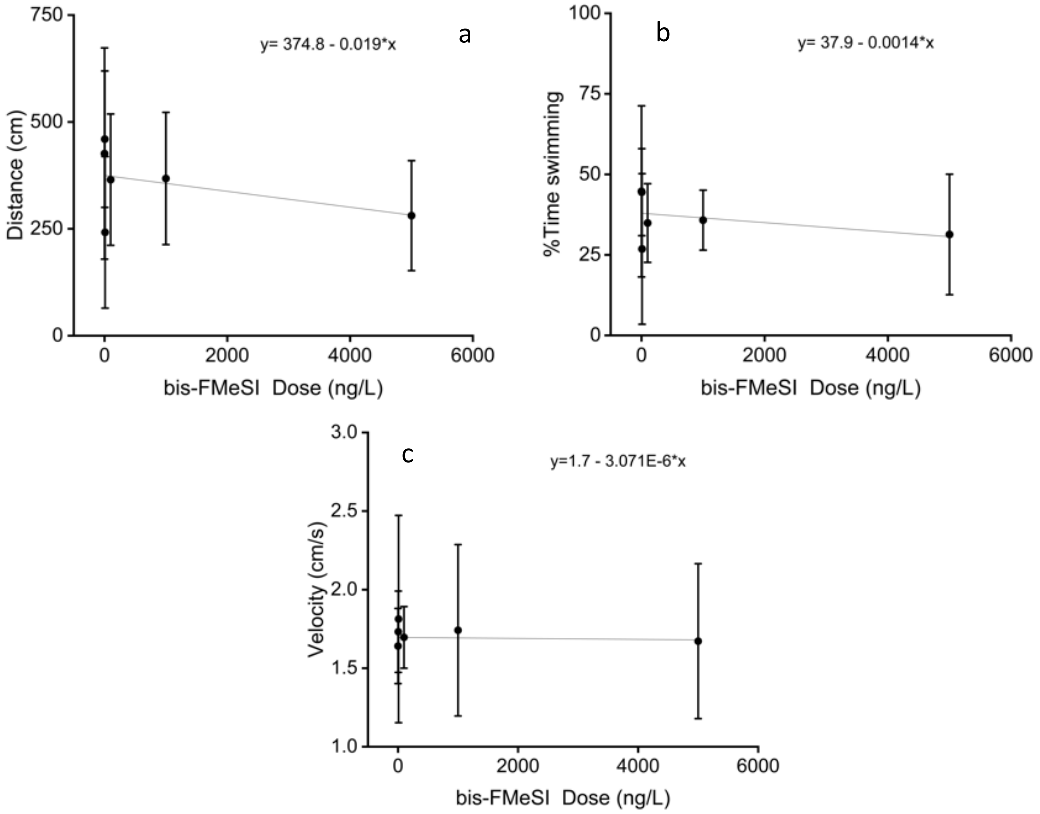

Supplementary Figure 15. Evaluation of individual swimming metrics vs. dose for *D. magna*. Pearson's correlations for distance (a), percent time swimming (b), and velocity (c) were -0.45 (p=0.238), -0.36 (p=0.320), and -0.32 (p=0.978), respectively, which indicates moderate to weak assocations that are not significant. These weaker associations result from high variability observed in each endpoint, including controls. Note that n=10 *D. magna* per dose except 5 ng L^{-1} (n=7), 1000 ng L^{-1} and 5000 ng L^{-1} (n=9) where organisms were immobilized or died prior to data collection. Error bars represent ± the standard error of the mean. Bis-FMeSI = bis-perfluoromethanesulfonimide. Source data can be found in **Supplementary Data 8**.

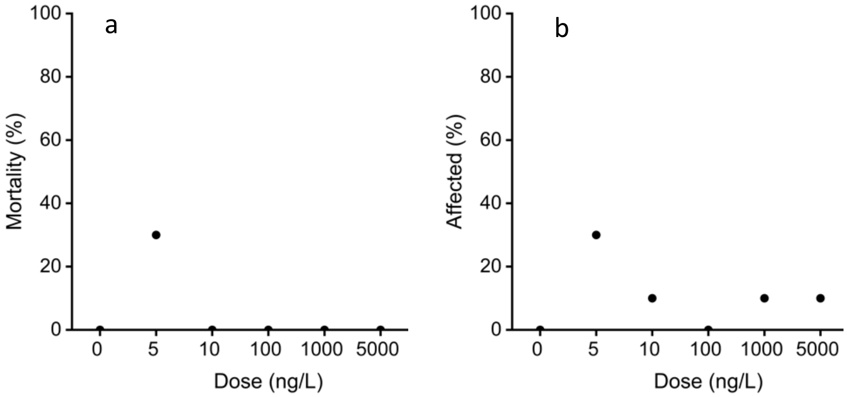

Supplementary Figure 16. Acute toxicity endpoints of (a) lethality and (b) lethality and immobilization. Ten total daphnia were exposed individually, therefore no standard deviation of either metric is reported. Test design was optimized for determination of swimming behavior. Source data are in **Supplementary Table 6**.

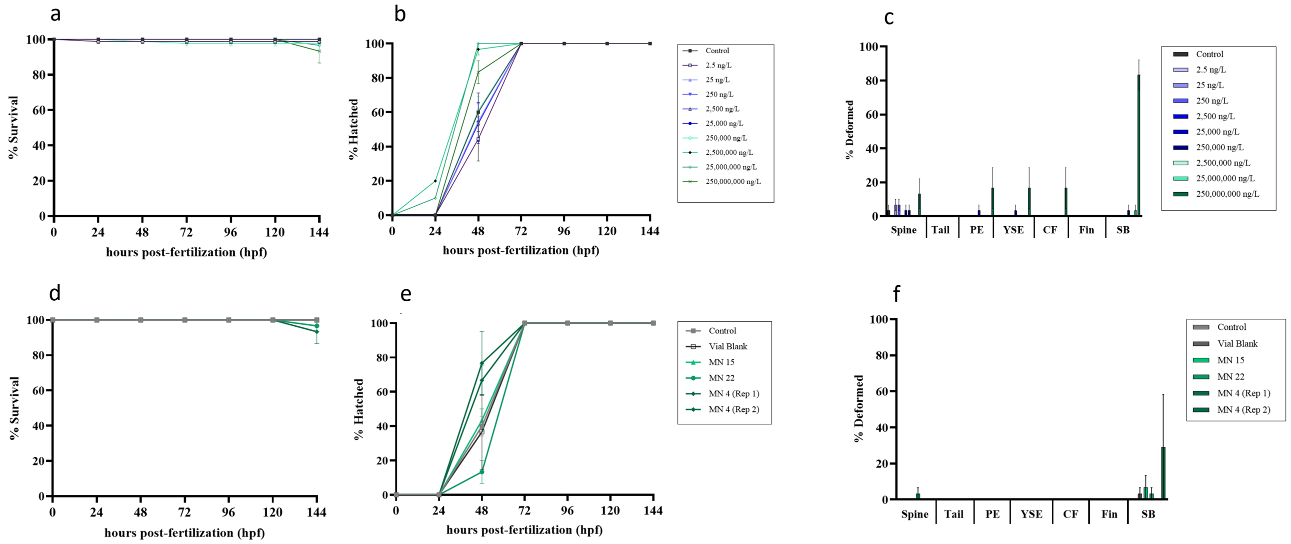

Supplementary Figure 17. Acute toxicity endpoints in zebrafish larvae exposed to bis-perfluoromethanesulfonimide (bis-FMeSI) (a-c) or field collected water samples (d-f) including survival (a,d), hatching rate (b,e), and developmental deformities (c,f). Points and bars represent means ± SEM. Abbreviations for types of developmental deformities: Spine – curvature of the spine; Tail – bending shortening, or alteration of the caudal tail; PE – pericardial edema; YSE – yolk sac edema; CF – craniofacial deformity; Fin – alteration of the pectoral fin or fin fold; SB – uninflated or less inflated swim bladder. n=90 for treatment groups ≤250,000 ng/L; n=30 for treatment groups ≥2,500,000 ng/L. No statistical differences between any groups (Kruskal-Wallis test; p>0.05) Source data can be found in **Supplementary Data 10-15**.

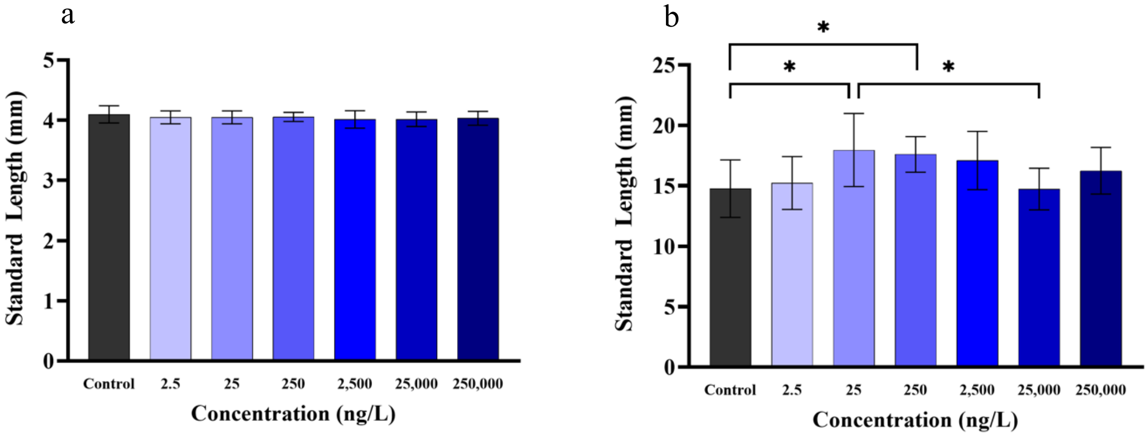

Supplementary Figure 18. Growth of zebrafish exposed to bis-perfluoromethanesulfonimide (bis-FMeSI). Standard lengths (mm) at 6 days post-fertilization (a) and 6 weeks post-fertilization (b). Bars represent means ± the standard error of the mean (SEM). Statistical differences between groups (*n*=30/treatment at 6dpf; *n*=7-16/treatment 6wpf; one-way ANOVA with post-hoc Tukey test; $p<0.05$). Source data can be found in **Supporting Data 15 and 16**.

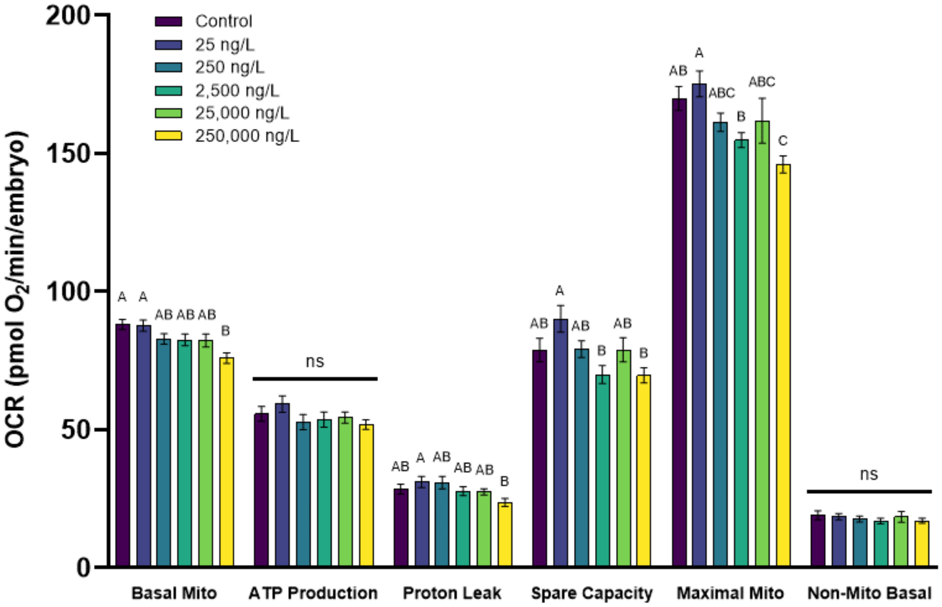

Supplementary Figure 19. Mitochondrial function of embryos (30 hpf) exposed to a range of concentrations of lithium bis-perfluoromethanesulfonimide. Histogram is grouped by mitochondrial parameter. Bars represent mean ±SEM. Different letters (e.g., A, AB, ABC) represent statistical differences within each parameter ($p<0.05$; one-way ANOVA or Kruskal-Wallis; *n*=28 zebrafish embryos for basal mitochondrial respiration, ATP Production, and non-mitochondrial respiration; *n*=14 zebrafish embryos for proton leak, spare capacity, and maximum mitochondrial respiration). Source data are available in **Supporting Data 17-19** and individual *p* values are available in **Supporting Data 20.**

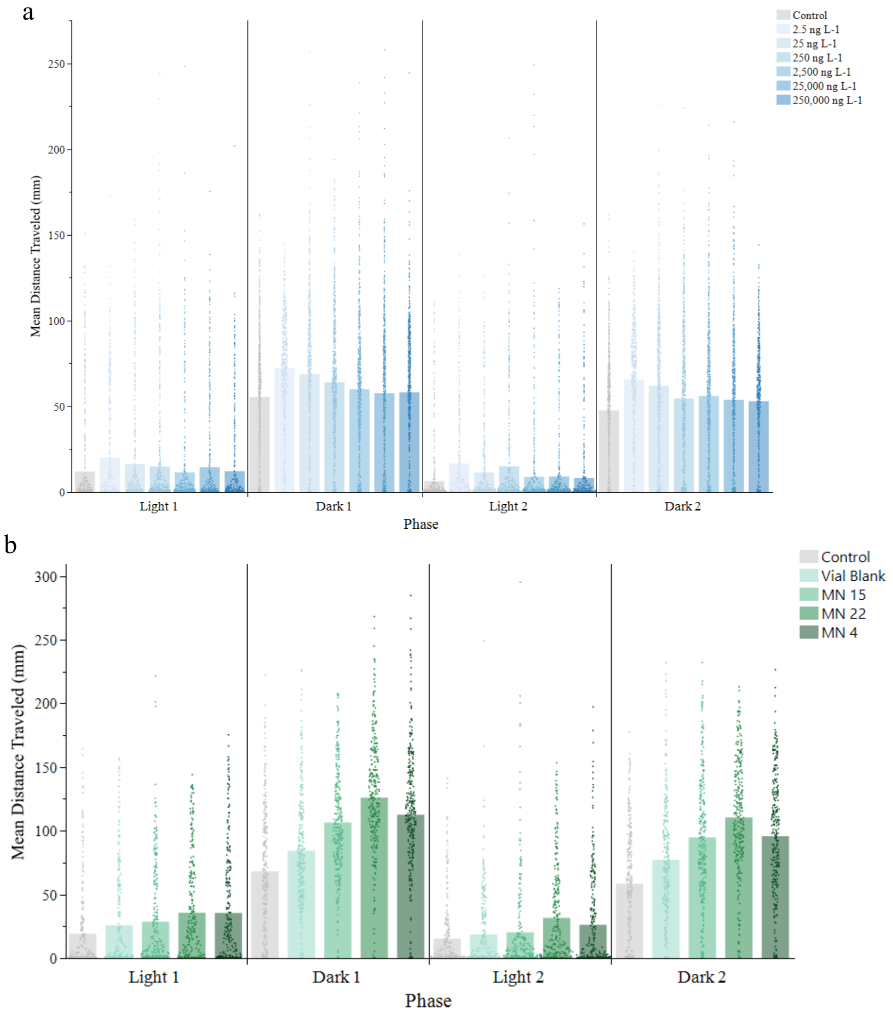

Supplementary Figure 20. Mean distance traveled (mm) during four, 10 min phases of the assay (two light and two dark) by larvae exposed to 25-250,000 ng L^{-1} bis(trifluoromethylsulfonyl)imide (bis-FMeSI) (a) and field-collected aqueous samples MN 4 (including duplicate), MN 22, and MN 15 (b). This is a version of Figures 3a and 3b from the main manuscript with corresponding data points overlaying the bar charts. Data points obscured the error bars and intercomparison results depicted in Figures 3a and 3b, so a separate version with data points is provided here. Source data can be found in **Supplementary Data 22-24**.

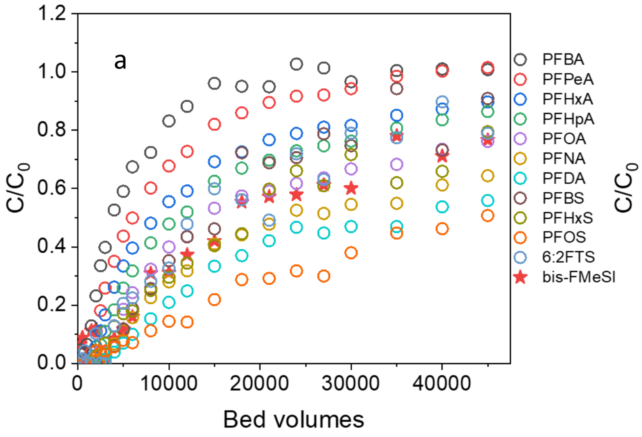

Supplementary Figure 21. Normalized breakthrough (C/C_0) curves of 21 per- and polyfluoroalkyl substances (PFAS) in the granular activated carbon column in coagulated, settled surface water with a total organic carbon level of 2.3 mg/L. Bis-perfluoromethanesulfonimide [bis-FMeSI] compared to perfluoroalkyl carboxylates and perfluoroalkyl sulfonates (a) and bis-FMeSI compared to per and polyfluoroalkyl ether acids (b). Acronyms for individual PFAS are in **Supporting Table 1**. Source data are available in **Supporting Data 18**.

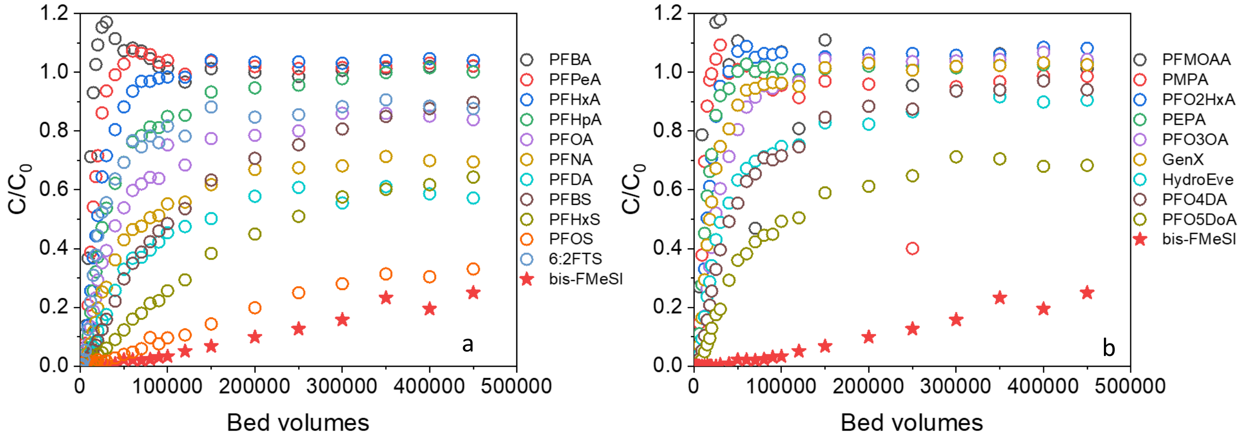

Supplementary Figure 22. Normalized breakthrough (C/C_0) curves of 21 21 per- and polyfluoroalkyl substances (PFAS) in the ion exchange resin column in groundwater with a total organic carbon level of 4.6 mg/L. Bis-perfluoromethanesulfonimide [bis-FMeSI] compared to perfluoroalkyl carboxylates and perfluoroalkyl sulfonates (a) and bis-FMeSI compared to per and polyfluoroalkyl ether acids (b). Acronyms for individual PFAS are in **Supporting Table 1**. Source data are available in **Supporting Data 18**.

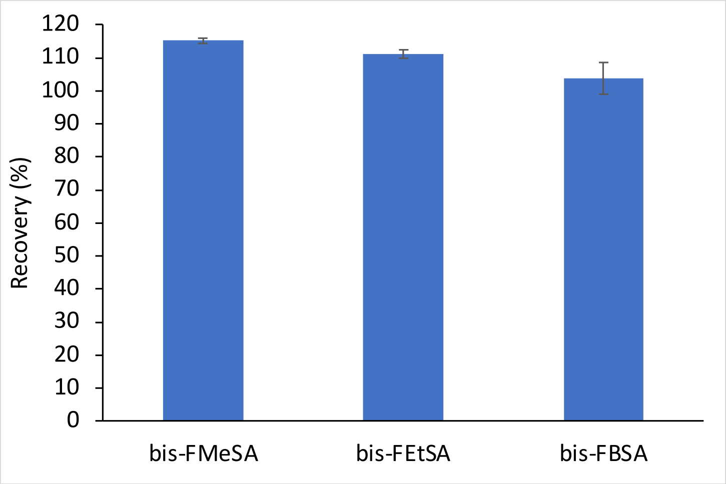

Supplementary Figure 23. Percent recovery of bis-perfluoromethanesulfonimide (bis-FMeSI), bis-perfluoroethanesulfonimide (bis-FEtSI), and bis-perfluorobutanesulfonimide (bis-FBSI) in spiked, deionized water following alkaline, heat activated persulfate activation. Error bars represent ± the standard error of the mean (SEM) based on analytical triplicates. Source data are available in **Supplementary Table 8**.

Supplementary Table 1. Target analytes and isotopically labeled standards used in this study.

Target analyte	Acronym	Isotopically labeled standards	CASRN
**Perfluoroalkyl carboxylate (PFCA)[1]**			
Perfluorobutanoic acid	PFBA	[$^{13}C_3$] PFBA	375-22-4
Perfluoropentanoic acid	PFPeA	[$^{13}C_4$] PFPeA	2706-90-3
Perfluorohexanoic acid	PFHxA	[$^{13}C_5$] PFHxA	307-24-4
Perfluoroheptanoic acid	PFHpA	[$^{13}C_4$] PFHpA	375-85-9
Perfluorooctanoic acid	PFOA	[$^{13}C_8$] PFOA	335-67-1
Perfluorononanoic acid	PFNA	[$^{13}C_8$] PFNA	375-95-1
Perfluorodecanoic acid	PFDA	[$^{13}C_6$] PFDA	335-76-2
Perfluoroundecanoic acid	PFUnA	[$^{13}C_7$]PFUnA	2058-94-8
Perfluorododecanoic acid	PFDoA	[$^{13}C_2$] PFDoA	307-55-1
Perfluorotridecanoic acid	PFTrDA	[$^{13}C_2$] PFTeDA	72629-94-8
Perfluorotetradecanoic acid	PFTeDA	[$^{13}C_2$] PFTeDA	376-06-7
**Perfluoroalkyl sulfonate (PFSA)[1]**			
Perfluorobutane sulfonic acid	PFBS	[$^{13}C_3$] PFBS	375-73-5
Perfluoropentane sulfonic acid	PFPeS	[$^{13}C_3$] PFBS	2706-91-4
Perfluorohexane sulfonic acid	PFHxS	[$^{13}C_3$]PFHxS	355-46-4
Perfluoroheptane sulfonic acid	PFHpS	[$^{13}C_8$] PFOS	375-92-8
Perfluorooctane sulfonic acid	PFOS	[$^{13}C_8$] PFOS	1763-23-1
Perfluorononane sulfonic acid	PFNS	[$^{13}C_8$] PFOS	68259-12-1
Perfluorodecane sulfonic acid	PFDS	[$^{13}C_8$] PFOS	335-77-3
**Per- and polyfluoroalkylether carboxylate (PFECA)[2]**			
Perfluoro-2-propoxypropanoic acid	GenX (HFPO-DA)	[$^{13}C_3$]-HFPO-DA	13252-13-6
4,8-dioxa-3H-perfluorononanoic acid	ADONA	[$^{13}C_8$] PFOA	919005-14-4
Perfluoro-2-methoxyacetic acid	PFMOAA	[$^{13}C_3$] PFBA	674-13-5
Perfluoro-3-methoxypropanoic acid (linear)	PFMPA[3]	[$^{13}C_3$] PFBA	377-73-1
Perfluoro-2-(perfluoromethoxy)propanoic acid (branched)		[$^{13}C_3$]-HFPO-DA	13140-29-9
Perfluoro-4-methoxybutanoic acid (linear)	PFMBA[4]	[$^{13}C_5$] PFHxA	863090-89-5
Perfluoro-2-ethoxypropanoic acid (branched)		[$^{13}C_3$]-HFPO-DA	267239-61-2
Perfluoro-3,5-dioxahexanoic acid	PFO2HxA	[$^{13}C_4$] PFPeA	39492-88-1
Perfluoro-3,6-dioxaheptanoic acid	PFO2HpA	[$^{13}C_4$] PFHpA	151772-58-6
Perfluoro-3,5,7-trioxaoctanoic acid	PFO3OA	[$^{13}C_5$] PFHxA	39492-89-2
Perfluoro-3,5,7,9-butaoxadecanoic acid	PFO4DA	[$^{13}C_4$] PFHpA	39492-90-5
Perfluoro-3,5,7,9,11-pentaoxadodecanoic acid	PFO5DoA	[$^{13}C_8$] PFNA	39492-91-6
Perfluoro-2-ethoxypropanoic acid	PEPA	[$^{13}C_4$] PFPeA	267239-61-2
HydroEve	HydroEve	[$^{13}C_4$] PFHpA	773804-62-9
**Per- and polyfluoroalkylether sulfonate (PFESA)[2]**			
Perfluoro(2-((6-chlorohexyl)oxy) ethanesulfonic acid)	9Cl-PF3ONS	[$^{13}C_8$] PFOS	756426-58-1
11-chloroeicosafluoro-3-oxaundecane-1-sulfonic acid	11Cl-PF3OUdS	[$^{13}C_8$] PFOS	763051-92-9
Nafion Byproduct 2	Nafion Byproduct 2	[$^{13}C_3$]PFHxS	749836-20-2
1,1,2,2-tetrafluoro-2-(1,2,2,2-tetrafluoroethoxy) ethanesulfonate	NFHOS	[$^{13}C_3$] PFBS	1132933-86 -8
**Chlorinated perfluoroalkyl sulfonate (Cl-PFSA)**			
Perfluoro-8-chloro-1-octanesulfonic acid	8Cl-PFOS	[$^{13}C_8$] PFOS	777011-38-8

**Fluorotelomer sulfonate (n:2 FtS)**			
4:2 fluorotelomer sulfonate	4:2 FTS	[$^{13}C_2$] 4:2FTS	757124-72-4
6:2 fluorotelomer sulfonate	6:2 FTS	[$^{13}C_2$] 6:2FTS	27619-97-2
8:2 fluorotelomer sulfonate	8:2 FTS	[$^{13}C_2$] 8:2FtS	39108-34-4
**Fluorotelomer carboxylate (n:x FTCA)**			
6:2 fluorotelomer carboxylic acid	6:2 FTCA	[$^{13}C_2$] 6:2FTCA	53826-12-3
8:2 fluorotelomer carboxylic acid	8:2 FTCA	[$^{13}C_2$] 8:2FTCA	27854-31-5
10:2 fluorotelomer carboxylic acid	10:2 FTCA	[$^{13}C_2$] 10:2FTCA	53826-13-4
3:3 fluorotelomer carboxylic acid	3:3 FTCA	[$^{13}C_2$] 6:2FTCA	356-02-5
5:3 fluorotelomer carboxylic acid	5:3 FTCA	[$^{13}C_2$] 6:2FTCA	914637-49-3
7:3 fluorotelomer carboxylic acid	7:3 FTCA	[$^{13}C_2$] 6:2FTCA	812-70-4
**Perfluoroalkanesulfonamide (FASA)**			
Perflurobutanesulfonamide	FBSA	[$^{13}C_8$] FOSA	30334-69-1
Perfluorohexanesulfonamide	FHxSA	[$^{13}C_8$] FOSA	41997-13-1
Perfluorooctanesulfonamide	FOSA	[$^{13}C_8$] FOSA	754-91-6
Perfluorodecanesulfonamide	FDSA	[$^{13}C_8$] FOSA	4262-70-8
**Bisperfluoroalkanesulfonimide (bisFASIs)**			
Bisperfluoromethane sulfonimide	bis-FMeSI	[$^{13}C_4$] PFPeA	82113-65-3
Bisperfluoroethanesulfonimide	bis-FEtSI	[$^{13}C_2$] PFHxA	152894-10-5
Bisperfluorobutanesulfonimide	bis-FBSI	[$^{13}C_4$] PFHpA	39847-39-7
**Perfluoroalkane sulfonamido acetic acid**			
N-methylperfluorooctanesulfonamido acetic acid	N-MeFOSAA	[$^{2}H_3$] N-MeFOSAA	2355-31-9
N-ethylperfluorooctanesulfonamido acetic acid	N-EtFOSAA	[$^{2}H_5$] N-EtFOSAA	2991-50-6

[1.]PFCAs and PFSAs referred to collectively as perfluoroalkyl acids (PFAAs). [2.]PFECAs and PFESAs are referred to collectively as per- and polyfluoroalkyl ether acids (PFEAs). [3.]PFMPA is used herein to refer to both the linear and branched isomer (sometimes referred to as PMPA). Use of the PMPA acronym was avoided because it can refer to multiple PFAS with different molecular weights and because the linear standard was used for analysis at TTU and the branched standard at Duke and NCSU. [4.]Consistent with PFMPA, PFMBA is used herein to refer to both linear and branched isomer (sometimes referred to as PEPA). The linear standard was used for analysis at TTU and the branched standard at Duke and NCSU.

Supplementary Table 2. Sample locations, dates, and types collected near Cottage Grove, MN.

Site Number	Site Description	Sample Date	Sample Matrix	Latitude	Longitude
MN 1	MS River Prescott public landing	1/2022	SW	44.74510	-92.79918
		6/2022	SW		
		6/2022	Sed		
MN 2	MS River @ Gey Cloud Dunes Nat'l Area	1/2022	SW	44.78764	-92.96211
		6/2022	SW		
MN 3	MS River @ lock & dam no. 2	1/2022	SW	44.75883	-92.87151
		6/2022	SW		
MN 4	3M outfall creek	1/2022	SW	44.78329	-92.89336
		6/2022	SW		
		6/2022	Sed		
MN 5	MS River @ Jaycee Park boat launch	1/2022	SW	44.74850	-92.85914
		6/2022	SW		
MN 6	Snow from MS River near 3M	1/2022	Snow	44.78449	-92.91171
MN 7	MS River upstream of 3M 1	1/2022	SW	44.78375	-92.90600
MN 8	MS River upstream of 3M 2	1/2022	SW	44.78343	-92.90568
		6/2022	SW		
MN 9	Downstream of Municipal WWTP	1/2022	SW	44.78475	-92.91514
		6/2022	SW		
MN 10	MS River @ lock & dam no. 2 outflow	1/2022	SW	44.75733	-92.86581
		6/2022	SW		
MN 11	MS River upstream of municipal WWTP	1/2022	SW	44.78469	-92.92147
		6/2022	SW		
MN 12	Archer Science	1/2022	*TW*	45.02794	-92.85572
		6/2022	*TW*		
MN 13	Private Home Well	1/2022	*GW*	44.77016	-92.79580
		6/2022	*GW*		
MN 14	Lower St. Croix River River	1/2022	SW	44.78173	-92.79408
		6/2022	SW		
MN 15	MS River @ kayak launch	6/2022	SW	44.79111	-92.97917
MN 16	Stillwater at old bridge	6/2022	SW	45.05750	-92.80444
MN 17	Office water bubbler	6/2022	*TW*	NA	NA
MN 18	MS River upstream of 3M @ barn house	6/2022	SW	44.78250	-92.94667
MN 19	MS River downstream of 3M @ railroad bridge & stream	6/2022	SW	44.77472	-92.87861
MN 20	MS River @ railroad bridge near dam	6/2022	SW	44.76472	-92.86889
MN 21	MS River @ Spring Lake Park boat launch	6/2022	SW	44.75889	-92.94000
MN 22	MS River @ confluence with 3M outfall creek	6/2022	SW	44.78306	-92.89333
MN 23	Tablyn Park Creek	6/2022	SW	44.99222	-92.92750
MN 24	Raleigh Creek	6/2022	SW	44.97806	-92.90306
		6/2022	Sed		
MN 25	Stillwater Private Home	6/2022	*TW*	45.06611	-92.80806
MN 26	Woodbury Private Home	6/2022	*TW*	45.90306	-92.08667
MN 27	Lake Elmo	6/2022	SW	44.98639	-92.88611
		6/2022	Sed		
MN 28	Pine Coulee Park	6/2022	Soil	44.77861	-92.86806
MN 29	3M Fenceline	6/2022	Soil	44.80333	-92.92333
MN 30	Wilson Park	6/2022	Soil	44.74194	-92.85417
MN 31	River Oaks Golf Course	6/2022	Soil	44.77861	-92.86806

Definitions: Surface water (SW); sediment (sed); tap water (TW); ground water (GW); Samples in *italics* represent drinking water sources

Supplementary Table 3. Sample locations, dates, and types collected near Paducah and Louisville, KY.

Site Number	Site Description	Sample Date	Sample Matrix	Latitude	Longitude
KY 1	TN @ Knoxville, TN	9/22/22	SW	35.92833	-83.95806
KY 2	Cumberland River @ Nashville, TN	9/22/22	SW	36.21694	-86.70528
KY 3	OH @ Downstream Paducah Transect West	9/23/22	SW	37.09250	-88.59611
KY 4	OH @ Downstream Paducah Transect Middle	9/23/22	SW	37.09556	-88.59444
KY 5	OH @ Downstream Paducah Transect East	9/23/22	SW	37.09694	-88.58861
KY 6	TN @ Downstream OH TN Converge	9/23/22	SW	37.06917	-88.56500
KY 7	OH @ Downstream OH TN Converge	9/23/22	SW	37.07167	-88.56417
KY 8	TN @ Downstream US 60 Bridge	9/23/22	SW	37.03361	-88.52639
KY 9	TN @ Downstream Paint Facility	9/23/22	SW	37.02694	-88.46083
KY 10	TN @ Downstream Recycling Facility	9/23/22	SW	37.05472	-88.39139
KY 11	TN @ between Outfalls 1 and 2	9/23/22	SW	37.06028	-88.37306
KY 12	TN @ Arkema Outfall 1	9/23/22	SW	37.05861	-88.37667
		9/23/22	Sed		
KY 13	TN @ Arkema Outfall 2	9/23/22	SW	37.06194	-88.35972
		9/23/22	Sed		
KY 14	TN @ Arkema Downstream Outfall 1	9/23/22	SW	37.05806	-88.37694
KY 15	TN @ I69 Bridge Upstream	9/23/22	SW	37.02583	-88.28694
KY 16	TN @ Dam Upstream	9/23/22	SW	37.01500	-88.27333
KY 17	OH @ Downstream of Arkema and Chemours	9/24/22	SW	38.20306	-85.87389
KY 18	OH @ Potential Arkema water intake	9/24/22	SW	38.21667	-85.85389
KY 19	OH @ Arkema Outfall	9/24/22	SW	38.21500	-85.85639
KY 20	OH @ Arkema Outfall beach	9/24/22	SW	38.21472	-85.85583
		9/24/22	Sed		
KY 21	OH @ Upstream of Arkema and Chemours	9/24/22	SW	38.22250	-85.84722
KY 22	OH @ Louisville Boat Ramp & Duke Energy	9/24/22	SW	38.26278	-85.83306
KY 23	Arkema Soil	9/24/22	Soil	37.05261	-88.36759
KY 24	SE of Arkema Soil	9/24/22	Soil	37.04944	-88.36333
KY 25	CCMA Metals	9/24/22	Soil	37.05500	-88.35194
KY 26	Recycling Facility	9/24/22	Soil	37.05194	-88.39306
KY 27	Paris Road	9/24/22	Soil	37.07028	-88.37833
KY 28	Richards Road	9/24/22	Soil	37.07417	-88.37083
KY 29	Fred Tracy Road	9/24/22	Soil	37.06389	-88.34472
KY 30	Haddock Ferry Road	9/24/22	Soil	37.06417	-88.35361
		9/24/22	Sed		
KY 31	OH @ Huntington, WV boat ramp	9/25/22	SW	38.42528	-82.44083
KY 32	Kanawa River @ South Charleston, WV boat ramp	9/25/22	SW	38.36139	-81.71694

Definitions: Surface water (SW); sediment (sed)

Supplementary Table 4. Sample locations, dates, and types collected near Antwerp, Belgium and Salindres, France

Site Number	Site Description	Sample Date	Sample Matrix	Latitude	Longitude
EU 1	Scheldt River @ Dutch border	10/24/22	SW	51.38444	4.20222
		10/24/22	Sed		
EU 2	Scheldt River @ electrical plant	10/24/22	SW	51.31861	4.26833
EU 3	Scheldt River @ mouth of shipping channel 1	10/24/22	SW	51.30417	4.27167
EU 4	Scheldt River @ mouth of shipping channel 2	10/24/22	SW	51.28417	4.25250
EU 5	Scheldt River @ Lanxess	10/24/22	SW	51.28583	4.32472
EU 6	Scheldt River @ Katoen Natie bend	10/24/22	SW	51.28417	4.31833
EU 7	Scheldt River @ Total (has outfall)	10/24/22	SW	51.27083	4.31250
EU 8	Scheld River @ downstream of 3M near DEME group	10/24/22	SW	51.25139	4.31472
EU 9	Scheldt River @ possible 3M outfall 1	10/24/22	SW	51.23861	4.34833
		10/24/22	Sed		
EU 10	Scheldt River @ possible 3M outfall 2	10/24/22	SW	51.23833	4.34806
		10/24/22	Sed		
EU 11	Scheldt River @ E34 bridge	10/24/22	SW	51.20639	4.36972
EU 12	Burcht @ BP oil	10/24/22	SW	51.19944	4.35083
EU 13	Scheldt @ boat dock	10/24/22	SW	51.22361	4.39472
EU 14	Soil in forest E of 3M	10/24/22	Soil	51.22889	4.36361
EU 15	Soil @ 3M fence line	10/24/22	Soil	51.23000	4.34194
EU 16	Blokkorsdijk Lake	10/24/22	SW	51.22944	4.34333
		10/24/22	Sed		
EU 17	Ditch near 3M	10/24/22	SW	51.22833	4.34639
		10/24/22	Sed		
EU 18	Bospolder parking lot	10/25/22	Soil	51.27556	4.33333
EU 19	Rivierenhof	10/25/22	Soil	51.21806	4.47028
EU 20	L'Arias far upstream	10/26/22	SW	44.17306	4.13944
		10/26/22	Sed		
		10/26/22	Soil		
EU 21	L'Avene far upstream	10/26/22	SW	44.18556	4.15639
		10/26/22	Sed		
		10/26/22	Soil		
EU 22	L'Avene upstream	10/26/22	SW	44.17778	4.15250
		10/26/22	Sed		
		10/26/22	Soil		
EU 23	L'Avene downstream	10/26/22	SW	44.15472	4.14861
		10/26/22	Sed		
		10/26/22	Soil		
EU 24	L'Arias at outfall	10/26/22	SW	44.16306	4.14306
		10/26/22	Sed		
EU 25	L'Arias downstream of merge with L'Avene	10/26/22	SW	44.14139	4.14694
		10/26/22	Sed		
		10/26/22	Soil		

Definitions: Surface water (SW); sediment (sed)

Supplementary Table 5. Parameters of GAC and IX columns used in this study.

Parameters	GAC	IX
Particle size (mm)	0.069	0.070
GAC/IX mass (g)	0.042	0.011
Empty bed contact time (min)	0.056	0.0151
Bed depth (cm)	0.99	0.425
Bed diameter (cm)	0.318	0.318
Flow rate (mL/min)	1.4	2.23
Hydraulic loading rate (m/h)	10.6	16.9
Total organic carbon concentration in the influent water (mg/L)	2.3	4.6
Total bed volume treated	50,000	500,000

Definitions: Granular activated carbon (GAC); ion exchange resin (IX)

Supplementary Table 6. Experimental water quality and bis-FMeSI dose confirmation data for all *D. magna* exposure testing and data for mortality and immobility. Water quality and parameters for EPA moderately hard water and measured values in dilution water for all 48-hr acute *D. magna* exposures.

Water Quality Parameter Validation		
Parameter	Method Requirements	Measured values
pH	7.4 - 7.8	7.5
Hardness (mg/L)	80 - 100	91
Alkalinity (mg/L)	57 - 64	62
DO (mg/L)	> 6 mg/L	7.68
Conductivity (µS/cm)	-	241
bis-FMeSI exposure dose validation		
Nominal (ng/L)		Measured ± SD (ng/L)
0		BQL
5		13.6 ±3.7
10		16.4 ±2.4
100		151 ±15.2
1000		1292 ±65.2
5000		6590 ±53.4
Lethality Results		
Nominal (ng/L)		No. organisms/10
0		0
5		3
10		0
100		0
1000		0
5000		0
Immobility Results		
Nominal (ng/L)		No. organisms/10
0		0
5		3
10		1
100		0
1000		1
5000		1

Definitions: bis-perfluoromethanesulfonimide (bis-FMeSI); dissolved oxygen (DO); below the limit of quantification (BQL)

Supplementary Table 7. Analysis of bis-FASIs in LiBs, battery binding agents, and landfill leachates

		bis(perfluoroalkyl)sulfonimides			
		**bis-FMeSI**	**bis-FEtSI**	**bis-FBSI**	
		Total mass in battery (± SD), ng			Battery type & size
**Batteries**	1. iPad Air battery A1484, 8827 mAh, 3.73 V	n.d.	n.d.	n.d.	LiPo, 133 mm (w) x 189 mm (h) 209 g
	2. iPhone 13 battery A2655, 3227 mAh, 3.84 V	n.d.	n.d.	n.d.	LiPo, 45 mm (w) x 88 mm (h) 80 g
	3. Liter Energy 102050 LiPo, 1000 mAh, 3.7 V	54.8 (6.3)	6.9 (0.1)	5.4 (0.3)	LiPo, 20 mm (w) x 50 mm (h), 19 g
	4. UltraFire, 1800 mAh, 3.7 V	21,800 (5,400)	n.d.	n.d.	14500, 14.2 mm (d) x 48.5 mm (h), 21 g
	5. Tesla battery, 3500 mAh, 3.7 V	n.d	13.3 (6.4)	n.d	
	6. Panasonic NCR-18650-B, 3400 mAh, 3.6 V	n.d.	5.4 (0.1)	n.d	
	7. Panasonic NCR-18650-GA, 3450 mAh, 3.6 V	19.3 (0.7)	n.d.	n.d.	
	8. Samsung ICR18650-30B, 2950 mAh, 3.7 V	35,600,000 (1,000,000)	n.d.	n.d.	18650, 18 mm (d) x 65 mm (h), 47 g
	9. Samsung INR-18650-30Q, 3000 mAh, 3.6 V	7.2 (1.6)	n.d.	n.d.	
	10. Samsung INR-18650-25R, 2500 mAh, 3.6 V	n.d	n.d.	n.d.	
	11. LithiumWerks APR-18650-M1B, 1200 mAh, 3.3 V	228.2 (1.2)	n.d.	n.d.	
	12. FST 18650, 2200 mAh, 3.6 V	8,290 (47)	n.d.	n.d.	
	13. Molicel INR-21700-P42, 4200 mAh, 3.6 V	n.d.	n.d.	n.d.	
	14. Samsung INR-21700-40T, 4000 mAh, 3.6 V	n.d.	n.d.	n.d.	
	15. Samsung INR-21700-50E, 5000 mAh, 3.6 V	29.1 (0.7)	n.d.	n.d.	21700, 21.7 mm (d) x 70.2 mm (h), 70 g
	16. Epoch INR-21700, 5000 mAh, 3.7 V	8.6 (0.2)	n.d.	n.d.	
	17. LG INR-21700-M50LT, 5000 mAh, 3.6 V	n.d.	n.d.	n.d.	
		Concentration in bulk material (± SD), ng/g			Description
	PVDF Binder	385 (2)	n.d.	n.d.	CAS 24937-79-9, avg. MW ~1,000,000
		Dissolved concentration, ng/L			Sample collection date
**Landfill leachate**	Leachate A	195	n.d	n.d.	1/10/22
		518	n.d	n.d.	3/25/22
		342	n.d	n.d.	4/7/22
		374	n.d	n.d.	5/24/22
	Leachate B	881	n.d	n.d.	1/10/22
		510	n.d	n.d.	3/25/22
		598	n.d	n.d.	4/7/22
		825	n.d	5.3	5/24/22

Definitions: bis-perfluoroalkyl sulfonamides (bis-FASIs); bis-perfluoromethanesulfonimide (bis-FMeSI); perfluoroethanesulfonimide (bis-FEtSI); bis-perfluorobutanesulfonimide (bis-FBSI); non-detect (n.d.); polyvinylidene fluoride (PVDF)

Supplementary Table 8. Concentrations of bis-FASIs in spiked, deionized water before and after alkaline, heat-activated persulfate oxidation.

Compound	Pre- oxidation (ng/L)	±	Post-oxidation (ng/L)	±	Recovery (%)
bis-FMeSI	862	13.9	994	6.67	115
bis-FEtSI	969	8.33	1080	13.2	111
bis-FBSI	652	36.7	676	31.3	104

Definitions: bis-perfluoroalkyl sulfonamides (bis-FASIs); bis-perfluoromethanesulfonimide (bis-FMeSI); perfluoroethanesulfonimide (bis-FEtSI); bis-perfluorobutanesulfonimide (bis-FBSI)

Supplementary References

1. Wang, Z. *et al.* A New OECD Definition for Per- and Polyfluoroalkyl Substances. *Environ. Sci. Technol.* **55**, 15575–15578 (2021).
2. Buck, R. C. *et al.* Perfluoroalkyl and Polyfluoroalkyl Substances in the Environment: Terminology, Classification, and Origins. *Integrated Environmental Assessment and Management* **7**, 513–541 (2011).
3. USEPA. *Drinking Water Contaminant Candidate List - Final. 40 CFR Part 141* vol. EPA–HQ–OW–2018–0594; FRL–7251–02– OW (2022).
4. USEPA. EPA: Chemical Contaminants - CCL 5 PFAS subset. (2022).
5. Arthur, S. D. *et al.* Nonaqueous electrolyte compositions comprising lithium oxalato phosphates. (2022).
6. 360 Research Reports. *Global LiTFSI Market Growth 2023-2029*. 92 (2023).
7. Arkema USA. ARKEMA HPP, PVDF Electrode Binders & Separator Coatings. https://hpp.arkema.com/en/markets-and-applications/renewable-energy/lithium-ion-battery/ (2022).
8. Howells, R. D., Lamanna, W. M., Fanta, A. D. & Waddell, J. E. Preparation of bis (fluoroalkylenesulfonyl) imides and (fluoroalkysulfony) (fluorosulfonyl) imides. (1999).
9. 3M. *Product Information: FluoradTM Lithium (Bis) Trifluoromethanesulfonimide Battery Electrolyte HQ-115*. (2002).
10. 3M. 3M Antistatic Additives. https://multimedia.3m.com/mws/media/1180156O/3m-antistatic-additives-overview-presentation.pdf (2016).
11. Solvay. *LiTFSi: Lithium Salt for Safe and Performing Batteries*. https://www.google.com/url?sa=t&rct=j&q=&esrc=s&source=web&cd=&ved=2ahUKEwjLisbPppH9AhWIfDABHWgSCtIQFnoECBMQAQ&url=https%3A%2F%2Fwww.solvay.com%2Fen%2FdownloadDocument%3FfileId%3DT5Yk9C04xcifrk18Yo%26fileName%3D24998%2520LiTFSI%2520for%2520Safe%2520and%2520Performing%2520Batteries%2520v2%26base%3DFAST&usg=AOvVaw1FZa-96nMjQ8KDJ3tSgkWa (NA).
12. Solvay. Manufactured products: Solvay Salindres Plant. https://www.solvay.fr/implantations/salindres/produits-fabriques (2023).
13. Solvay. Solvay accelerates its growth in specialty fluoroaliphatic derivatives for electronics and pharmaceuticals markets. https://www.solvay.com/en/press-release/solvay-accelerates-its-growth-specialty-fluoroaliphatic-derivatives-electronics-and (2013).
14. Schmidt, G. Use of lithium salt mixtures as li-ion battery electrolytes. (2015).
15. Arkema. Kynar PVDF Provides High Purity, Chenically Resistant Solutions for the Semiconductor Industry. https://hpp.arkema.com/en/markets-and-applications/chemical-industry-and-general-industry/chemical-process-industry-overview/semiconductor-industry/ (2022).
16. Waddell, J. E., Lamanna, W. M., Krause, L. J., Moore, G. G. I. & Hamrock, S. J. Perfluoroalkylsulfonates, sulfonimides, and sulfonyl methides, and electrolytes containing them. (1996).
17. Guo, Y. *et al.* Ionic liquids with two typical hydrophobic anions as acidic corrosion inhibitors. *Journal of Molecular Liquids* **269**, 886–895 (2018).
18. Taccardi, N. *et al.* Catalyst recycling in monophasic Pt-catalyzed hydrosilylation reactions using ionic liquids. *Applied Catalysis A: General* **399**, 69–74 (2011).

19. Weiß, A. *et al.* Modification of nitrogen doped carbon for SILP catalyzed hydroformylation of ethylene. *Catal. Sci. Technol.* **7**, 5562–5571 (2017).
20. Kulkarni, P. S., Ranjane, P., Mishra, K., Sundararajan, S. & Kamble, S. Tetraalkylammonium-based dicationic ionic liquids (ILs) for CO_2 capture. *New J. Chem.* **47**, 12944–12954 (2023).
21. Sandhu, S., Kaur, N., Kaur, M. & Singh, V. Performance of {111}-TiO2/betanin/[Emim][NTf2]/Ag dye sensitized solar cell – combined experimental and DFT studies. *Materials Letters* **312**, 131717 (2022).
22. USEPA. *ORD Human Health Toxicity Value for Lithium Bis[(Trifluoromethyl)Sulfonyl]Azanide (HQ-115)*. https://nepis.epa.gov/Exe/ZyPDF.cgi/P10188AG.PDF?Dockey=P10188AG.PDF (2023).
23. ECHA. Lithium bis(trifluoromethylsulfonyl)imide Ecotoxicological Summary. https://echa.europa.eu/sk/registration-dossier/-/registered-dossier/18080/6/1 (2020).
24. Neuwald, I. J. *et al.* Efficacy of activated carbon filtration and ozonation to remove persistent and mobile substances – A case study in two wastewater treatment plants. *Science of The Total Environment* **886**, 163921 (2023).
25. Davis, K. L., Aucoin, M. D., Larsen, B. S., Kaiser, M. A. & Hartten, A. S. Transport of ammonium perfluorooctanoate in environmental media near a fluoropolymer manufacturing facility. *Chemosphere* **67**, 2011–2019 (2007).
26. Schroeder, T., Bond, D. & Foley, J. PFAS soil and groundwater contamination *via* industrial airborne emission and land deposition in SW Vermont and Eastern New York State, USA. *Environ. Sci.: Processes Impacts* **23**, 291–301 (2021).
27. Strynar, M. *et al.* Identification of Novel Perfluoroalkyl Ether Carboxylic Acids (PFECAs) and Sulfonic Acids (PFESAs) in Natural Waters Using Accurate Mass Time-of-Flight Mass Spectrometry (TOFMS). *Environmental Science & Technology* **49**, 11622–11630 (2015).
28. Sun, M. *et al.* Legacy and Emerging Perfluoroalkyl Substances Are Important Drinking Water Contaminants in the Cape Fear River Watershed of North Carolina. *Environmental Science & Technology Letters* **3**, 415–419 (2016).
29. Gaber, N., Bero, L. & Woodruff, T. J. The Devil they Knew: Chemical Documents Analysis of Industry Influence on PFAS Science. *Annals of Global Health* **89**, 37 (2023).
30. Anderson, B. G. The Toxicity Thresholds of Various Sodium Salts Determined by the Use of Daphnia magna. *Sewage Works Journal* **18**, 82–87 (1946).
31. Tkaczyk, A., Bownik, A., Dudka, J., Kowal, K. & Ślaska, B. Daphnia magna model in the toxicity assessment of pharmaceuticals: A review. *Science of The Total Environment* **763**, 143038 (2021).
32. Bownik, A. Daphnia swimming behaviour as a biomarker in toxicity assessment: A review. *Science of The Total Environment* **601–602**, 194–205 (2017).
33. Krewski, D. *et al.* Toxicity testing in the 21st century: progress in the past decade and future perspectives. *Arch Toxicol* **94**, 1–58 (2020).
34. Dionísio, R., Daniel, D., Alkimin, G. D. D. & Nunes, B. Multi-parametric analysis of ciprofloxacin toxicity at ecologically relevant levels: Short- and long-term effects on Daphnia magna. *Environmental Toxicology and Pharmacology* **74**, 103295 (2020).
35. Foguth, R., Sepúlveda, M. S. & Cannon, J. Per- and Polyfluoroalkyl Substances (PFAS) Neurotoxicity in Sentinel and Non-Traditional Laboratory Model Systems: Potential Utility in Predicting Adverse Outcomes in Human Health. *Toxics* **8**, 42 (2020).

36. Gaballah, S. *et al.* Evaluation of Developmental Toxicity, Developmental Neurotoxicity, and Tissue Dose in Zebrafish Exposed to GenX and Other PFAS. *Environ Health Perspect* **128**, 047005 (2020).
37. Rericha, Y. *et al.* Behavior Effects of Structurally Diverse Per- and Polyfluoroalkyl Substances in Zebrafish. *Chem. Res. Toxicol.* **34**, 1409–1416 (2021).
38. Ulhaq, M., Örn, S., Carlsson, G., Morrison, D. A. & Norrgren, L. Locomotor behavior in zebrafish (Danio rerio) larvae exposed to perfluoroalkyl acids. *Aquatic Toxicology* **144–145**, 332–340 (2013).
39. Hagenaars, A., Vergauwen, L., Benoot, D., Laukens, K. & Knapen, D. Mechanistic toxicity study of perfluorooctanoic acid in zebrafish suggests mitochondrial dysfunction to play a key role in PFOA toxicity. *Chemosphere* **91**, 844–856 (2013).
40. Eschauzier, C., Beerendonk, E., Scholte-Veenendaal, P. & De Voogt, P. Impact of Treatment Processes on the Removal of Perfluoroalkyl Acids from the Drinking Water Production Chain. *Environmental Science & Technology* **46**, 1708–1715 (2012).
41. Rahman, M. F., Peldszus, S. & Anderson, W. B. Behaviour and fate of perfluoroalkyl and polyfluoroalkyl substances (PFASs) in drinking water treatment: A review. *Water Research* **50**, 318–340 (2014).
42. Houtz, E. F. & Sedlak, D. L. Oxidative Conversion as a Means of Detecting Precursors to Perfluoroalkyl Acids in Urban Runoff. *Environmental Science & Technology* **46**, 9342–9349 (2012).
43. Shojaei, M. *et al.* Enhanced Recovery of Per- and Polyfluoroalkyl Substances (PFASs) from Impacted Soils Using Heat Activated Persulfate. *Environ. Sci. Technol.* acs.est.0c08069 (2021) doi:10.1021/acs.est.0c08069.
44. Nzeribe, B. N., Crimi, M., Mededovic Thagard, S. & Holsen, T. M. Physico-Chemical Processes for the Treatment of Per- And Polyfluoroalkyl Substances (PFAS): A review. *Critical Reviews in Environmental Science and Technology* **49**, 866–915 (2019).
45. Belkouteb, N., Franke, V., McCleaf, P., Köhler, S. & Ahrens, L. Removal of per- and polyfluoroalkyl substances (PFASs) in a full-scale drinking water treatment plant: Long-term performance of granular activated carbon (GAC) and influence of flow-rate. *Water Research* **182**, 115913 (2020).
46. Pan, Y. *et al.* Worldwide Distribution of Novel Perfluoroether Carboxylic and Sulfonic Acids in Surface Water. *Environ. Sci. Technol.* **52**, 7621–7629 (2018).
47. Dennis, N. M. *et al.* Chronic Reproductive Toxicity Thresholds for Northern Bobwhite Quail (*Colinus virginianus*) Exposed to Perfluorohexanoic Acid (PFHxA) and a Mixture of Perfluorooctane Sulfonic Acid (PFOS) and PFHxA. *Enviro Toxic and Chemistry* **40**, 2601–2614 (2021).
48. Dykstra, C. R., Route, W. T. & Williams, K. A. Trends and Patterns of Perfluoroalkyl Substances in Blood Plasma Samples of Bald Eagle Nestlings in Wisconsin and Minnesota, USA. *Environ Toxicol Chem* **40**, 754–766 (2021).
49. Barzen-Hanson, K. A. *et al.* Discovery of 40 Classes of Per- and Polyfluoroalkyl Substances in Historical Aqueous Film-Forming Foams (AFFFs) and AFFF-Impacted Groundwater. *Environmental Science & Technology* **51**, 2047–2057 (2017).
50. Nikinmaa, M. & Anttila, K. Individual variation in aquatic toxicology: Not only unwanted noise. *Aquatic Toxicology* **207**, 29–33 (2019).

51. Nikinmaa, M., Suominen, E. & Anttila, K. Water-soluble fraction of crude oil affects variability and has transgenerational effects in Daphnia magna. *Aquatic Toxicology* **211**, 137–140 (2019).
52. Szabó, B., Lang, Z., Kövér, S. & Bakonyi, G. The inter-individual variance can provide additional information for the ecotoxicologists beside the mean. *Ecotoxicology and Environmental Safety* **217**, 112260 (2021).
53. Orlando, F. E. & Guillette, J. L. A re-examination of variation associated with environmentally stressed organisms. *APMIS* **109**, S178–S186 (2001).
54. Tkaczyk, A., Bownik, A., Dudka, J., Kowal, K. & Ślaska, B. Daphnia magna model in the toxicity assessment of pharmaceuticals: A review. *Science of The Total Environment* **763**, 143038 (2021).
55. Bownik, A. Daphnia swimming behaviour as a biomarker in toxicity assessment: A review. *Science of The Total Environment* **601–602**, 194–205 (2017).
56. Steudte, S., Stepnowski, P., Cho, C.-W., Thöming, J. & Stolte, S. (Eco)toxicity of fluoro-organic and cyano-based ionic liquid anions. *Chem. Commun.* **48**, 9382 (2012).
57. Labine, L. M. *et al.* Sublethal Exposure of Per- and Polyfluoroalkyl Substances of Varying Chain Length and Polar Functionality Results in Distinct Metabolic Responses in *Daphnia magna*. *Enviro Toxic and Chemistry* **42**, 242–256 (2023).
58. Labine, L. M. *et al.* Comparison of sub-lethal metabolic perturbations of select legacy and novel perfluorinated alkyl substances (PFAS) in Daphnia magna. *Environmental Research* **212**, 113582 (2022).
59. USEPA. *Methods for Measuring the Acute Toxicity of Effluents and Receivng Waters to Freshwater and Marine Organisms, Fifth Edition.* https://www.epa.gov/sites/default/files/2015-08/documents/acute-freshwater-and-marine-wet-manual_2002.pdf (2002).
60. Hazlerigg, C. R. E., Lorenzen, K., Thorbek, P., Wheeler, J. R. & Tyler, C. R. Density-Dependent Processes in the Life History of Fishes: Evidence from Laboratory Populations of Zebrafish Danio rerio. *PLoS ONE* **7**, e37550 (2012).
61. Brand, M. D. & Nicholls, D. G. Assessing mitochondrial dysfunction in cells. *Biochemical Journal* **435**, 297–312 (2011).
62. Chacko, B. K. *et al.* The Bioenergetic Health Index: a new concept in mitochondrial translational research. *Clinical Science* **127**, 367–373 (2014).
63. Meyer, J. N., Hartman, J. H. & Mello, D. F. Mitochondrial Toxicity. *Toxicological Sciences* **162**, 15–23 (2018).
64. Fichi, G. *et al.* Fishing in the Cell Powerhouse: Zebrafish as A Tool for Exploration of Mitochondrial Defects Affecting the Nervous System. *IJMS* **20**, 2409 (2019).
65. Lowery, L. A., De Rienzo, G., Gutzman, J. H. & Sive, H. Characterization and Classification of Zebrafish Brain Morphology Mutants. *The Anatomical Record* **292**, 94–106 (2009).
66. Huang, M. *et al.* Developmental and mitochondrial toxicity assessment of perfluoroheptanoic acid (PFHpA) in zebrafish (Danio rerio). *Environmental Toxicology and Pharmacology* **97**, 104037 (2023).
67. Patel, N., Ivantsova, E., Konig, I., Souders, C. L. & Martyniuk, C. J. Perfluorotetradecanoic Acid (PFTeDA) Induces Mitochondrial Damage and Oxidative Stress in Zebrafish (Danio rerio) Embryos/Larvae. *Toxics* **10**, 776 (2022).
68. Arp, H. P. H. & Hale, S. E. Assessing the Persistence and Mobility of Organic Substances to Protect Freshwater Resources. *ACS Environ. Au* **2**, 482–509 (2022).

69. Guelfo, J. L. & Higgins, C. P. Subsurface Transport Potential of Perfluoroalkyl Acids at Aqueous Film-Forming Foam (AFFF)-Impacted Sites. *Environmental Science & Technology* **47**, 4164–4171 (2013).
70. Steudte, S. *et al.* Hydrolysis study of fluoroorganic and cyano-based ionic liquid anions – consequences for operational safety and environmental stability. *Green Chem.* **14**, 2474 (2012).
71. Liu, J. & Mejia Avendaño, S. Microbial degradation of polyfluoroalkyl chemicals in the environment: A review. *Environment International* **61**, 98–114 (2013).
72. Hale, S. E., Arp, H. P. H., Schliebner, I. & Neumann, M. Persistent, mobile and toxic (PMT) and very persistent and very mobile (vPvM) substances pose an equivalent level of concern to persistent, bioaccumulative and toxic (PBT) and very persistent and very bioaccumulative (vPvB) substances under REACH. *Environ Sci Eur* **32**, 155 (2020).
73. Droge, S. T. J. Membrane–Water Partition Coefficients to Aid Risk Assessment of Perfluoroalkyl Anions and Alkyl Sulfates. *Environ. Sci. Technol.* **53**, 760–770 (2019).
74. Dołżonek, J. *et al.* Membrane partitioning of ionic liquid cations, anions and ion pairs – Estimating the bioconcentration potential of organic ions. *Environmental Pollution* **228**, 378–389 (2017).
75. Shojaei, M., Kumar, N. & Guelfo, J. L. An Integrated Approach for Determination of Total Per- and Polyfluoroalkyl Substances (PFAS). *Environ. Sci. Technol.* **56**, 14517–14527 (2022).
