# Supplementary material for: Lithium-ion battery components are at the nexus of sustainable energy and environmental release of per- and polyfluoroalkyl substances
Source: Nat Commun. 2024 Jul 8;15:5548. doi: 10.1038/s41467-024-49753-5 (PMC11231300; doi:10.1038/s41467-024-49753-5)
Supplement: Supplementary file 3 — Description of Additional Supplementary Files [file 41467_2024_49753_MOESM3_ESM.pdf]

Description of Additional Supplementary Files

Supplementary Data Legend:

Supplementary Data 1. Concentrations of PFAS measured in surface water and snow (MN6) samples during the January 2022 sampling event in the Cottage Grove, MN region. PFAS from Table S1 that are not shown here were not detected in any project field sample.

Supplementary Data 2. Concentrations of PFAS measured in surface water samples during the June 2022 sampling event in the Cottage Grove, MN region. PFAS from Table S1 that are not shown here were not detected in any project field sample.

Supplementary Data 3. Concentrations of PFAS measured in soil (MN 28-MN 31) and sediment (MN1, MN4, MN24, MN 27) samples during the June 2022 sampling event in the Cottage Grove, MN region. PFAS from Table S1 that are not shown here were not detected in any project field sample.

Supplementary Data 4. Concentrations of PFAS measured in surface water samples collected while traveling to and from KY (KY 1-2, KY 31-32) near Paducah, KY (KY 2-KY1 6) and near Louisville, KY (KY 17-22) during the September 2022 sampling event. PFAS from Table S1 that are not shown here were not detected in any project field sample.

Supplementary Data 5. Concentrations of PFAS measured in sediment (KY 12-13, KY20, KY30) and soil (KY 27-29) near Paducah, KY and Louisville, KY (KY 20 only) during the September 2022 sampling event. PFAS from Table S1 that are not shown here were not detected in any project field sample.

Supplementary Data 6. Concentrations of PFAS measured in surface water samples near Antwerp (EU 1 - 17) and Salindres (EU 24 - 26) during the September 2022 sampling event.PFAS from Table S1 that are not shown here were not detected in any project field sample.

Supplementary Data 7. Concentrations of PFAS measured in sediment (denoted "sed") in Antwerp, Belgium (EU 1 - EU 19) and Salindres, France (EU 20 - EU 25) during the October 2022 sampling event. PFAS from Table S1 that are not shown here were not detected in any project field sample.

Supplementary Data 8. Swimming metrics for individual replicates in the D. Magna exposures.

Supplementary Data 9. Summary table of p-values when comparing mean distance traveled and time spent by daphnia neonates exposed to bis-FMeSI in the acute toxicity assay. Values in top are from distance traveled and values in bottom are from the percentage of time spent swimming. Bolded values $P<0.05$; Italicized values $P<0.1$

Supplementary Data 10. Zebrafish survival rate (%) from 0-144 hpf during exposure to bis-FMeSI; data represented as proportions of survival within each technical replicate (n=10 individuals/technical replicate)

Supplementary Data 11. Zebrafish survival rate (%) from 0-144 hpf during exposure to field collected water samples

Supplementary Data 12. Zebrafish developmental deformities (%) at 6 dpf during exposure to bis-FMeSI; data represented as proportions of changed individuals within each technical replicate (n=10 individuals/technical replicate)

Supplementary Data 13. Zebrafish developmental deformities (%) at 6 dpf during exposure to bis-FMeSI

Supplementary Data 14. Zebrafish hatching rate (%) from 0-144 hpf during exposure to bis-FMeSI; data represented as proportions of hatched individuals within each technical replicate (n=10 individuals/technical replicate)

Supplementary Data 15. Zebrafish hatching rate (%) from 0-144 hpf during exposure to field collected water samples

Supplementary Data 16. Zebrafish standard length (mm) at 6 dpf following exposure to bis-FMeSI

Supplementary Data 17. Zebrafish standard length (mm) at 6 wpf following exposure to bis-FMeSI until 6dpf and then grown out in clean conditions

Supplementary Data 18. Bis-FMeSI Mitochondria Oligo-SA

Supplementary Data 19. Bis-FMeSI Mitochondria FCCP-SA

Supplementary Data 20. Summary table for mitochondrial parameters in zebrafish embryos exposed to bis-FMeSI

Supplementary Data 21. Summary table of p-values when comparing for mitochondrial parameters in zebrafish embryos exposed to bis-FMeSI. A) Values in top right are for basal mitochondrial respiration and values in bottom left are for non-mitochondrial respiration.

Supplementary Data 22. Locomotion data for zebrafish exposed to bis-FMeSI

Supplementary Data 23. Locomotion data for zebrafish exposed to field-collected samples from Pool 2 of the Mississippi River.

Supplementary Data 24. Summary table for mean distances traveled by zebrafish larvae in the locomotion assay

Supplementary Data 25. Summary table of p-values when comparing mean distance traveled by zebrafish larvae exposed to bis-FMeSI in the locomotion assay. Values in top right are from the first phase and values in bottom left are from second period of light/dark. Bolded values $P<0.05$; Italicized values $P<0.1$

Supplementary Data 26. Summary table of p-values when comparing mean distance traveled by zebrafish larvae exposed to field-collected water samples in the locomotion assay. Values in top right are from the first phase and values in bottom left

Supplementary Data 27. Coefficients of variation for each experimental bis-FMeSI group in each phase of the zebrafish locomotion assay

Supplementary Data 28. Coefficients of variation for each experimental water sample group in each phase of the zebrafish locomotion assay

Supplementary Data 29. Nominal and measured concentrations of bis-FMeSI in zebrafish exposure water

Supplementary Data 30. Relative concentrations (C/C0) of bis-FMeSI, PFOA, and PFOS measured during rapid small scale column tests with a. granular activated carbon (GAC) and b. ion exchange (IX) resin

Supplementary Data 31. Relative concentrations (C/C0) of 21 PFAS measured during rapid small scale column tests with granular activated carbon (GAC) in coagulated, settled surface water with total organic carbon = 2.3 mg/L

Supplementary Data 32. Relative concentrations (C/C0) of 21 PFAS measured during rapid small scale column tests with ion exchange resin (IX) in groundwater with total organic carbon = 4.6 mg/L
