# Supplementary material for: Lithium-ion battery components are at the nexus of sustainable energy and environmental release of per- and polyfluoroalkyl substances
Source: Nat Commun. 2024 Jul 8;15:5548. doi: 10.1038/s41467-024-49753-5 (PMC11231300; doi:10.1038/s41467-024-49753-5)
Supplement: Supplementary file 5 — Reporting Summary [file 41467_2024_49753_MOESM5_ESM.pdf]

Corresponding author(s): Jennifer L. Guelfo
P. Lee Ferguson

Last updated by author(s): 04/28/2024

Reporting Summary

Nature Portfolio wishes to improve the reproducibility of the work that we publish. This form provides structure for consistency and transparency in reporting. For further information on Nature Portfolio policies, see our Editorial Policies and the Editorial Policy Checklist.

Statistics

For all statistical analyses, confirm that the following items are present in the figure legend, table legend, main text, or Methods section.

n/a	Confirmed	
☐	☒	The exact sample size (*n*) for each experimental group/condition, given as a discrete number and unit of measurement
☐	☒	A statement on whether measurements were taken from distinct samples or whether the same sample was measured repeatedly
☐	☒	The statistical test(s) used AND whether they are one- or two-sided<br>*Only common tests should be described solely by name; describe more complex techniques in the Methods section.*
☐	☒	A description of all covariates tested
☐	☒	A description of any assumptions or corrections, such as tests of normality and adjustment for multiple comparisons
☐	☒	A full description of the statistical parameters including central tendency (e.g. means) or other basic estimates (e.g. regression coefficient) AND variation (e.g. standard deviation) or associated estimates of uncertainty (e.g. confidence intervals)
☐	☒	For null hypothesis testing, the test statistic (e.g. *F*, *t*, *r*) with confidence intervals, effect sizes, degrees of freedom and *P* value noted<br>*Give P values as exact values whenever suitable.*
☒	☐	For Bayesian analysis, information on the choice of priors and Markov chain Monte Carlo settings
☒	☐	For hierarchical and complex designs, identification of the appropriate level for tests and full reporting of outcomes
☐	☒	Estimates of effect sizes (e.g. Cohen's *d*, Pearson's *r*), indicating how they were calculated

Our web collection on statistics for biologists contains articles on many of the points above.

Software and code

Policy information about availability of computer code

Data collection	Sciex Operating System (OS) 2.2.05738 for high performance liquid chromatography (HPLC) mass spectrometry (MS) data collection (TTU); TraceFinder 5.1 (5.1.51.11) for liquid chromatography tander mass spectrometry (LC-MS/MS) data collection (Duke); Agilent MassHunter Work Station for LC-MS/MS data collection (NCSU); Ethovision XT 13 and JMP Pro 16 software for zebrafish data collection.
Data analysis	Sciex Operating System (OS) 2.2.05738 for HPLC-MS data processing; TSQ Altis Tune Application 3.2 (3.2.2625.33) for LC-MS/MS data collection (Duke); Agilent MassHunter Quantitative Analysis for LC-MS/MS data processing (NCSU). Ethovision XT 13 and JMP Pro 16 software for zebrafish data processing; Microsoft Excel 16.83, RStudio, OriginLab 2021b, OriginaPro2022b, GraphPad Prism 9.5.1 used for data calculations, statistical analysis, and creation of figures.

For manuscripts utilizing custom algorithms or software that are central to the research but not yet described in published literature, software must be made available to editors and reviewers. We strongly encourage code deposition in a community repository (e.g. GitHub). See the Nature Portfolio guidelines for submitting code & software for further information.

Data

Policy information about availability of data

All manuscripts must include a data availability statement. This statement should provide the following information, where applicable:

- Accession codes, unique identifiers, or web links for publicly available datasets
- A description of any restrictions on data availability
- For clinical datasets or third party data, please ensure that the statement adheres to our policy

All data associated with this sutdy are present in the paper or supplementary materials.

Research involving human participants, their data, or biological material

Policy information about studies with human participants or human data. See also policy information about sex, gender (identity/presentation), and sexual orientation and race, ethnicity and racism.

Reporting on sex and gender	This study does not include research involving human participants.
Reporting on race, ethnicity, or other socially relevant groupings	This study does not include research involving human participants.
Population characteristics	This study does not include research involving human participants.
Recruitment	This study does not include research involving human participants.
Ethics oversight	This study does not include research involving human participants.

Note that full information on the approval of the study protocol must also be provided in the manuscript.

Field-specific reporting

Please select the one below that is the best fit for your research. If you are not sure, read the appropriate sections before making your selection.

☐ Life sciences ☐ Behavioural & social sciences ☒ Ecological, evolutionary & environmental sciences

For a reference copy of the document with all sections, see nature.com/documents/nr-reporting-summary-flat.pdf

Ecological, evolutionary & environmental sciences study design

All studies must disclose on these points even when the disclosure is negative.

Study description

The Lethal and sublethal effects of bis-FMeSI on Daphnia magna (D. magna) was evaluated using a factorial design. D. magna exposed to bis-FMeSI following a modified 48 h acute, static non-renewal design described in EPA-821-R-02-01253. Ten D. magna were exposed in individual beakers to to 0, 5, 10, 100, 1000 and 5000 ng L-1 bis-FMeSI placed randomly in an environmental chamber at constant temperature and light conditions. At 48 hours each beaker was checked for dead or immobilized D. magna after which live organism swimming behavior was recorded. Still frames were analyzed to assess identify the swimming tracks of each individually exposed D. magna, and the sublethal endpoints of, swimming distance, time, and velocity were evaluated using this data.

Danio rerio (D. rerio) experiments were also conducted using a factorial design. At 6 hours post-fertilization (hpf), normal embryos were selected and transferred to small glass Petri dishes each containing 10 ml of treatment solution. Treatments consisted of bis-FMeSI dissolved in nanopure water to make a stock concentration of 0.48 mg/ml. This stock was diluted into test concentrations with 30% Danieau's medium. Each compound was tested at the following concentrations (ng/L): 0 (control) 25, 250, 2500, 25000, or 250000. Exposures were also conducted with field-collected water samples from the MN region. There was a final density of 1 embryo/ml/dish and three replicate dishes per treatment group (n 30) for each assay. Dishes were placed in an incubator at 28°C with a 14:10 h light:dark cycle. Embryos were observed daily for survival and hatching. Mortality was determined by lack of heartbeat, and ark cycle. Embryos were observed daily for survival and hatching. Mortality was determined by lack of heartbeat, and those individuals were removed from the dish. Embryos (n=14/group) were also assayed in vivo at 30 hpf for mitochondrial function. Larvae (n=60/group) were assayed at 144 hpf for behavior. A subset (n<20/group) was grown in clean, colony conditions until 4 mpf to determine growth.

Research sample

D. magna neonates (<24-h old) were obtained from Aquatic Biosystems (Ft Collins, CO) and used as received. D. magna were selected for use as this is a sensitive model organism for evaluating the toxicity to environmental receptors. Further, neonate organisms are tested to ensure toxicity information is reflective of the most sensitive life states of this organism. The use of this organism was established by the EPA as an appropriate test organism for whole effluent testing (WET).

Zebrafish (Danio rerio) are a prominent aquarium model fish in the study of human disease and environmental toxicology. As asynchronous fish with a short breeding cycle, embryos can be collected throughout the year at very specific stages of development.

The small size of zebrafish (adults ~5 cm in length, larvae ~0.5 cm in length) and their transparent embryos are useful traits for studying developmental toxicity. Its rapid development cycle (hatch at ~72 hours post fertilization) make it well suited to our experimental goals. Their wide use in the field of toxicology allows for the understanding of chemical accumulation, whole organism response, and localized tissue response. The wide array of tools provides ways to tie potential mechanisms to exposure. Their rapid development (48-72 hrs to hatching) also allows for rapid assessment developmental alterations and early life stage effects. These characteristics are useful for studies on environmental monitoring, and by deciphering the mechanisms of their toxicity in vertebrate fish models, we can extrapolate the findings and implications to human health.

Mitochondrial stressors early in life can impact brain development and the integrity of neural networks, causing cause behavioral changes. Chronic exposures can cause both structural and functional dysregulation. The developmental stage selected for mitochondrial function assays is the the Prim-15 stage (6 hpf) where the embryo has a relatively similar number of mitochondria relative to their body size compared to later stages of development including adults. This allows for the rapid assessment of mitochondrial (dys)function.

The locomotion assay takes advantage of instinctual behavioral responses with a visual stimulus using a light/dark transition test. As such, it can be measured at early, larval developmental stages. In this way, the assessment is rapid and because the animals are still very small, it increases the throughput of the assay.

Sampling strategy

D. magna: Data for all endpoints was collected 48 hours post exposure. Each beaker was checked for dead or immobilized D. magna after which live organism swimming behavior was recorded. Still frames were analyzed to identify the swimming tracks of each D. magna. Sublethal endpoints of swimming distance, time, and velocity were evaluated using this data. D. magna sample size was selected based on EPA-821-R-02-01253 and the technological capability to collect data (individual camera), number of organisms per treatment (10) and sampling time (10 min per organism). No sample size calculation was performed prior to data collection, however statistical power was calculated using obtained data for all sublethal endpoints assessed.

D. rerio: We used established sampling for aquatic toxicology. Technical replicates (triplicate dishes per treatment group) made up each of the biological replicates (repeats collected and tested on different days in the same laboratory) to capture variability and increase reproducibility.

Data collection

D. magna data was recorded as an mp4 file by A. Doria, and transferred to a laboratory computer to immediately back up files.

M. Chernick collected data associated with D. rerio exposures. D. rerio embryos (n=60/group) were assayed in vivo at 30 hpf for mitochondrial function using the Agilent Seahorse XFe96 Extracellular Flux Analyzer (Agilent Instruments, Santa Clara, CA). Oxygen consumption (OCR; pmol/min) was measured during a time course in which drugs are injected to in order to quantify various mitochondrial processes by modifying functions of the electron transport chain (ETC). Oligomycin A (9.4 µM; Sigma-Aldrich, St. Louis, MO) was injected following basal measurements to inhibit ATP synthase (complex V) and measure ATP-linked respiration. Proton leak was calculated based on the difference between ATP-linked respiration and basal respiration. Carbonyl cyanide 4 (trifluoromethoxy)phenylhydrazone (FCCP, 2.5 µM; Sigma-Aldrich) is an uncoupler that collapses the proton gradient and disrupts mitochondrial membrane potential. The resultant uninhibited flow of electrons through the ETC and oxygen consumption by complex IV pushes mitochondria to their maximum respiration rate. From this, maximum respiration and spare capacity (the difference between maximal and basal respiration), the ability to respond to an increased energy demand or stress, was calculated. Sodium azide (6.25 mM; Sigma-Aldrich) inhibits oxidative phosphorylation via inhibition of cytochrome c oxidase, rapidly depleting intracellular ATP and effectively shutting down mitochondrial respiration. The remaining non-mitochondrial respiration is attributed to enzymes and other factors within the cells that continue to consume oxygen.

Original zebrafish testing occurred in September 2022. We tested again in September 2023 to increase sample sizes and include an additional concentration as requested by a reviewer. Larval locomotion was measured at 6 days post-fertilization (dpf) using the DanioVision video-tracking system with Ethovision XT 13 software (Noldus, Leesburg, VA, USA). Larvae were individually transferred to wells of a clear 96-well plates (Greiner Bio-One, Monroe, NC) containing clean Danieau's medium (n=30/group). Then larvae were acclimated in the plate at 28°C for 1 hr. At the end of this acclimation period, the plate was placed in the DanioVision observation chamber 28°C. A 50-min long assay was then begun. The first 10 min of the assay was a habituation period in the dark to these conditions followed by two, alternating 10 min long light and dark periods. Larvae were assessed for total distance traveled (mm) within light and dark as well as the accumulated distance traveled during the assay not including the habituation period.

Timing and spatial scale

D. magna: D. magna data exposures were started on December 20th, and data was collected 48-hours after experiments were set up on December 22 2022. All exposure chambers were monitored for lethality and immobility at 24 hours, however, only 48-hour data is presented to be consistent with the exposure time period as specified in the standard method (EPA-821-R-02-01253). Lethality and immobility data was collected visually on a per organism basis while sublethal endpoints were assessed from swimming track data extracted from Mp4 videos were collected at a frame rate of 30fps.

Adult Ekkwill zebrafish were maintained iwith a 14:10 h light:dark cycle. Fish were fed Artemia nauplii in the mornings and Zeigler's Adult Zebrafish Complete Diet (Pentair Aquatic Ecosystems) in the afternoons. Breeder tanks, each containing 3 females and 2 males, were set up at 4 PM on the day before embryo exposures. Adults were spawned naturally the following morning within 2 hours of the beginning of the light cycle. Embryos were transferred to Petri dishes (VWR International, West Chester, PA, USA) containing 30% Danieau's medium (17.4 mM NaCl, 0.21 mM KCl, 0.12 mM $MgSO_4$, 0.18 mM $Ca(NO_3)_2$, and 1.5 mM HEPES, pH 7.2) and placed in an incubator at 28°C until exposure.

At 6 hours post-fertilization (hpf; shield stage), embryos were screened for viability and development. Normal embryos were selected and transferred to small glass Petri dishes each containing 10 ml of treatment solution. After exposure, D. rerio embryos (n=14/group) were assayed in vivo at 30 hpf for mitochondrial function. Larval locomotion was measured at 6 days post-fertilization. Larvae were individually transferred to wells of a clear 96-well plates (Greiner Bio-One, Monroe, NC) containing clean Danieau's medium (n=30/group). Then larvae were acclimated in the plate at 28°C for 1 hr. At the end of this acclimation period, the plate was placed in the DanioVision observation chamber 28°C. A 50-min long assay was then begun. The first 10 min of the assay was a habituation period in the dark to these conditions followed by two, alternating 10 min long light and dark periods.

Data exclusions	D. magna: Deceased or immobilized organisms were not included as this would have biased data by including organisms which could not swim. Additional outliers present in sublethal endpoints were identified using the The ROUT method (Robust regression and outlier removal). Data is first fitted with a non-linear regression to establish a base line that is used to remove outliers when Q-5%. Q value represents the false discovery rate, in other words, the proportion of false positives incurred by the method when the test is significant. This approach as selected for D. magna data collected here as it is appropriate for datasets characterized by high variability including non-normally distributed data.<br><br>D rerio: The Agilent Seahorse XF Analyzer takes repeated measurements of oxygen levels in the media (pmol/O2/min) and the oxygen probe may occasionally record a value that deviates from all other measurements for that individual within a measurement phase. Because the lowest or highest points within a phase are averaged for each individual to determine the value of a parameter, one outlier within a phase could influence and incorrectly skew these data. The method for identification and removal of outlier points was made by comparing each measurement for each individual to the measurements at the same time for all of the other individuals within the same treatment group. In this way, outliers were only determined within a treatment group so as to not remove a low/high measurement that was the result of a treatment effect. This method was applied to all data points equally and without bias.
Reproducibility	D. Magna: Ten organisms were exposed per treatment, including negative control exposures). The behavior of each individually exposed organism was measured for 10 min. No attempts to execute the experiments failed.<br><br>D. rerio: We did not have any of our experiments fail. All repeats of the experiment were successful.
Randomization	D. magna: All D. magna exposures were conducted in an environmental test chamber set to 20 ± 1°C with a 16-h light 8-h dark cycle. Despite the controlled exposure conditions, each beaker containing an individual D. magna was assigned to a position within the test chamber using a random number generator.<br><br>D. rerio: Adults were randomly selected from the fish colony for breeding. Resultant embryos were assessed for normal development and correct staging (i.e., 6hpf) and then randomly assigned to treatment dishes.
Blinding	The per- and polyfluoroalkyl substances (PFAS) data obtained in this study was collected using high performance liquid chromatography-mass spectrometry based approaches. Appropriate calibration, reference and check standards were implemented to eliminate bias due to factors such as matrix interference during data collection. Field collected samples used in D. rerio exposure testing were blinded to the analyst. Toxicity data collected for D. rerio and D. magna was extracted using code based algorithms and subsequently processed using the appropriate statistical methodology therefore removing bias, therefore blinding was not relevant.

Did the study involve field work? ☒ Yes ☐ No

Field work, collection and transport

Field conditions	Field conditions (Minneapolis-St. Paul; January 22, 2022): temperature ranged from -5 F to 7 F, conditions were sunny and wind was from the northwest at <10 miles per hour. Field conditions (Minneapolis-St. Paul; June 9, 2022): temperature ranged from 66 F to 81 F, conditions were sunny, and wind was from the west at 10 to 15 miles per hour. Field conditions (Paducah, KY; September 23, 2022): temperatures ranged from 60 F to 78 F and wind was from the wast at 5-10 miles per hour. Field conditions (louisville, KY; September 24, 2022): temperatures ranged from 60 F to 78 F and wind was from the south-southeast at 10-16 miles per hour. Field conditions (Antwerp, Belgium; October 24, 2022): temperatures ranged from 55 F to 63 F and winds were from the south-southwest at 7-12 miles per hour. Field conditions (Salindres, France; October 26, 2022): temperatures ranged from 64 F to 73 F and winds were from the northeast at 7 miles per hour.
Location	In general, samples were collected in rivers (MIssissippi, Ohio, Tennessee, and Scheldt Rivers) and streams (L'Arias and L'avene) that are or suspected to be receiving outfall discharges from major fluorochemical manufacturers (e.g., 3M, Arkema, and Solvay) and in the surrounding regions. In total, 31 surface water samples, 1 snow sample, 5 tap water samples, 2 groundwater samples, 4 sediment samples, and 4 soil samples were collected in January and June 2022 from 31 locations near Cottage Grove, MN (Table S2). Additionally, a total of 24 surface water samples, 4 sediment samples, and 8 soil samples were collected from 32 locations in Paducah, KY and Louisiville, KY (Table S3). Finally, a total of 21 surface water samples, 11 sediments samples, 9 soil samples, and 1 foam sample were collected from 25 locations in Antwerp, Belgium and Salindres, France in Ocrtober 2022 (Table S4). Tables S2-S4 contain the latitute and longitude information for all samples. All surface water samples were collected within the upper 1 ft of the water column.
Access & import/export	All samples were collected from publicly accessible locations. International soils were extracted on location and were not transported to the laboratories of the author team in the United States. There are no import restrictions on aqueous samples.
Disturbance	No disturbances resulted from this study.

Reporting for specific materials, systems and methods

We require information from authors about some types of materials, experimental systems and methods used in many studies. Here, indicate whether each material, system or method listed is relevant to your study. If you are not sure if a list item applies to your research, read the appropriate section before selecting a response.

Materials & experimental systems

n/a	Involved in the study
☒	☐ Antibodies
☒	☐ Eukaryotic cell lines
☒	☐ Palaeontology and archaeology
☐	☒ Animals and other organisms
☒	☐ Clinical data
☒	☐ Dual use research of concern
☒	☐ Plants

Methods

n/a	Involved in the study
☒	☐ ChIP-seq
☒	☐ Flow cytometry
☒	☐ MRI-based neuroimaging

Animals and other research organisms

Policy information about studies involving animals; ARRIVE guidelines recommended for reporting animal research, and Sex and Gender in Research

Laboratory animals	Daphnia magna neonates (<24 hours old) used here were obtained from a commercial toxicological organism supplier (Aquatic Biosystems). Routine QA/QC testing using EPA wet testing guidelines is performed by this supplier, so there is high confidence in the quality and strain of D. magna tested in this work.<br><br>The zebrafish strain was EkkWill. Testing was conducted on embryos 6 and 144 hours post fertilization.
Wild animals	This study did not involve wild animals.
Reporting on sex	D. magna: D magna are female and reproduce asexually in controlled laboratory cultures. Therefore all D. magna tested here are genetically identical.<br><br>D. rerio: We are not able to determine the sex of fish at the early stages tested, 6 hpf and 144 hpf. So we know it was a mix of males and females but we don't know the exact ratios.
Field-collected samples	There were no field-collected animals or research organisms.
Ethics oversight	No ethical oversight is required for D. magna. The D. rerio studies conducted in this manuscript were approved by Duke University Institutional Animal Care and Use Committee, Protocol Registry Number A069-22-04.

Note that full information on the approval of the study protocol must also be provided in the manuscript.

Plants

Seed stocks	No plants were used in this study.
Novel plant genotypes	No plants were used in this study.
Authentication	No plants were used in this study.
